# Calcitriol, calcidiol, parathyroid hormone, and fibroblast growth factor-23 interactions in chronic kidney disease

**DOI:** 10.1111/vec.12036

**Published:** 2013-04-08

**Authors:** Joao F Brito Galvao, Larry A Nagode, Patricia A Schenck, Dennis J Chew

**Affiliations:** VCA Arboretum View Animal HospitalDowners Grove, IL, 60515; Departments of Veterinary Biosciences; Clinical Sciences; College of Veterinary Medicine, The Ohio State UniversityColumbus, OH, 43210; Diagnostic Center for Population and Animal Health, Michigan State University4125 Beaumont Rd, Lansing, MI, 48910

**Keywords:** angiotensin-II, calcium, canine, feline, hyperparathyroidism, KLOTHO, phosphorus, RAAS, TACE, vitamin D

## Abstract

**Objective:**

To review the inter-relationships between calcium, phosphorus, parathyroid hormone (PTH), parent and activated vitamin D metabolites (vitamin D, 25(OH)-vitamin D, 1,25(OH)_2_-vitamin D, 24,25(OH)_2_-vitamin D), and fibroblast growth factor-23 (FGF-23) during chronic kidney disease (CKD) in dogs and cats.

**Data Sources:**

Human and veterinary literature.

**Human Data Synthesis:**

Beneficial effects of calcitriol treatment during CKD have traditionally been attributed to regulation of PTH but new perspectives emphasize direct renoprotective actions independent of PTH and calcium. It is now apparent that calcitriol exerts an important effect on renal tubular reclamation of filtered 25(OH)-vitamin D, which may be important in maintaining adequate circulating 25(OH)-vitamin D. This in turn may be vital for important pleiotropic actions in peripheral tissues through autocrine/paracrine mechanisms that impact the health of those local tissues.

**Veterinary Data Synthesis:**

Limited information is available reporting the benefit of calcitriol treatment in dogs and cats with CKD.

**Conclusions:**

A survival benefit has been shown for dogs with CKD treated with calcitriol compared to placebo. The concentrations of circulating 25(OH)-vitamin D have recently been shown to be low in people and dogs with CKD and are related to survival in people with CKD. Combination therapy for people with CKD using both parental and activated vitamin D compounds is common in human nephrology and there is a developing emphasis using combination treatment with activated vitamin D and renin-angiotensin-aldosterone-system (RAAS) inhibitors.

## Introduction

Renal secondary hyperparathyroidism (HPTH) is common in dogs and cats with chronic kidney disease (CKD). Complex interactions between circulating ionized calcium (iCa), inorganic phosphorus (Pi), parathyroid hormone (PTH), calcidiol (25(OH)-vitamin D), calcitriol (1,25(OH)_2_-vitamin D), and fibroblast growth factor 23 (FGF-23) occur during CKD. Relative and absolute deficits of the most biologically active vitamin D metabolite, calcitriol, are central in the genesis of renal secondary HPTH. Though not emphasized until very recently, deficits of calcidiol are also common in CKD and may contribute to renal secondary HPTH and other adverse systemic effects. Total body phosphorus burden and increasing concentration of circulating phosphorus play a pivotal role in the development of renal secondary HPTH and are intimately related to dynamics of calcitriol and FGF-23.

## Regulation of Calcium Metabolism

Regulation of serum calcium concentration is complex and requires the integrated actions of PTH, vitamin D metabolites, calcitonin, and iCa itself on calcium-sensing receptors. The following discussion is derived from several veterinary review articles over the past 20 years.^1^–^4^ The concentration of circulating iCa is tightly regulated as it is this fraction that exerts biological activity. The protein-bound and complexed fractions of circulating calcium are not specifically regulated and serve mainly as reservoirs for iCa in the circulation. PTH and calcitriol are the main regulators of calcium homeostasis. PTH is largely responsible for the minute-to-minute control of serum iCa concentration, whereas calcitriol maintains day-to-day control of serum iCa concentration. The intestine, kidney, and bone are the major target organs affected by calcium regulatory hormones. These interactions allow conservation of calcium in the extracellular fluid (ECF) volume by renal tubular reabsorption, increased intestinal transport of calcium from the diet, and internal redistribution of calcium from bone. The skeleton serves as a major supply of calcium and phosphorus when intestinal absorption and renal reabsorption are inadequate to maintain the normal serum calcium concentrations. Bone calcium mobilization is important in the acute regulation of blood calcium. Calcium and phosphorus can be mobilized from readily available calcium phosphate in the bone ECF compartment, but these stores are rapidly depleted. The osteoblast is critical in limiting the distribution of calcium and phosphate between bone and ECF, as exchangeable bone water is separated from ECF water by the combined membranes of osteoblasts lining bone surfaces. For greater or prolonged release of calcium from bone, osteoclastic bone resorption must be activated. Osteoclasts secrete acid and proteases that result in the dissolution of the mineralized matrix of bone and thus mobilize calcium and phosphorus. The extracellular iCa concentration is the fraction of total calcium that is actively regulated. When blood calcium concentration falls, PTH secretion is stimulated. PTH exerts direct effects on bone and kidney and indirect effects on the intestine through calcitriol. PTH increases synthesis of calcitriol by activating renal mitochondrial 1-α-hydroxylation of calcidiol derived from the circulation. Calcitriol, in turn, increases calcium absorption from the intestine. Calcitriol participates with PTH to stimulate osteoclastic bone resorption. Calcitriol is necessary for differentiation of osteoclasts from precursor mononuclear cells. At high pharmacologic doses, calcitriol can stimulate bone (without PTH) following actions on osteoblasts that subsequently liberate one or more factors that activate osteoclastic bone resorption. PTH increases osteoclast number and stimulates osteoclast function to increase bone resorption and the release of calcium from bone to blood. Calcitriol also induces renal transport mechanisms activated by PTH that increase tubular reabsorption of calcium from the glomerular filtrate, thus preventing calcium loss in urine. In addition, calcitriol exerts important inhibition of parathyroid gland (PTG) gene transcription for the synthesis of PTH.

## Vitamin D Compounds

**Figure 1:**
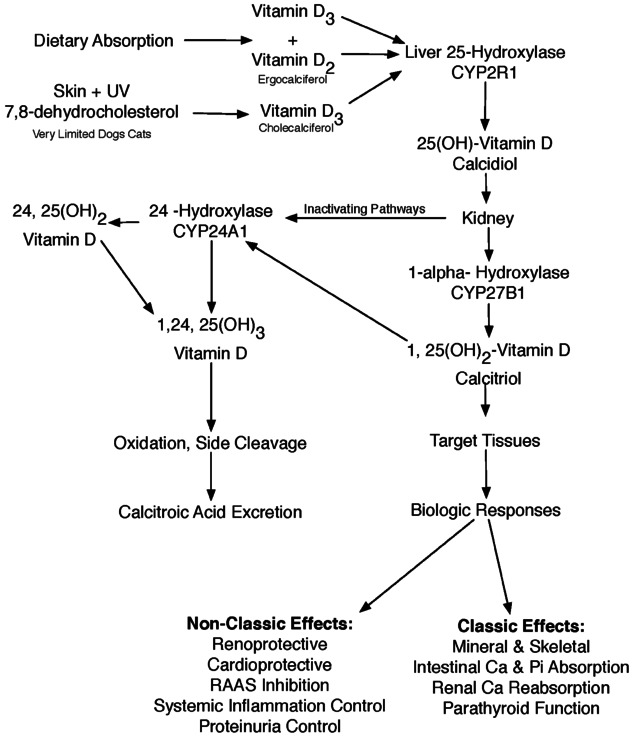
Vitamin D metabolic pathways.

Vitamin D is a prohormone^5^ that undergoes a two-step bioactivation to calcitriol as the most biologically active metabolite (Figure 1).^6^ The term vitamin D should be carefully used to refer to 1 of 2 calciferols, ergocalciferol (vitamin D_2_ of plant origin), or cholecalciferol (vitamin D_3_ synthesized in skin or from animal tissue). Vitamin D is sometimes referred to as vitamin D_2_/D_3_ to emphasize the different sources of this vitamin. It has been widely viewed that vitamin D_2_ and vitamin D_3_ are equipotent in people.^7^ This concept has been challenged in one human study in which a single high-dose of oral vitamin D_2_ resulted in less generation of circulating calcidiol than that following the same oral dose of vitamin D_3_; this effect was postulated likely to be due to less binding to circulating vitamin D binding protein (VDBP),^7^ which would deliver less vitamin D_2_ to the liver for biotransformation. In this study, vitamin D_2_ supplementation was from one-third to one-tenth as potent as that seen with vitamin D_3_ supplementation. Vitamin D_2_ based analogues appear to be less calcemic, an effect that may reflect greater catabolism of the unbound fraction of vitamin D_2_. In another study, daily oral dosing with vitamin D_2_ was equally effective in maintaining calcidiol concentration.^8^ Vitamin D_2_ is the only high-dose oral vitamin D compound approved by the Food and Drug Administration (FDA).^7^

## Vitamin D Metabolism

Vitamin D metabolites have poor aqueous solubility; thus they are bound in the circulation to the high-affinity transport protein known as VDBP. This carrier serves to limit the catabolism of vitamin D metabolites and also acts as a buffer to limit the action of the vitamin D metabolite.^6^ Biological activity of circulating vitamin D compounds is related to the free concentrations of these metabolites, but less than 1% of vitamin D compounds are free in the circulation. Very little native vitamin D exists in the body due to its rapid conversion under normal conditions to calcidiol. Vitamin D is stored in adipose tissue and continuously released for conversion to calcidiol during supraphysiologic intake.^9^ The vitamin D-VDBP complex is delivered to the liver where it undergoes the first step of bioactivation. There hydroxylation to calcidiol occurs in a poorly regulated process facilitated by the cytochrome P-450 25-hydroxylase (CYP2R1).^6^ This is a substrate-dependent process as circulating concentrations of calcidiol parallel the amount of vitamin D intake.

Calcidiol is the predominant circulating metabolite of vitamin D and reflects vitamin D status. The reference interval for serum circulating calcidiol is 62–88 nmol/L (25–35 ng/mL) in people. A calcidiol concentration from 75 to 90 ng/mL has been suggested to provide protection against some degenerative diseases in people.^5^ The upper limit of the reference interval for calcidiol in dogs and cats is considerably higher (60–215 nmol/L for dogs; 65–170 nmol/L for cats),^4^ most likely due to their routine consumption of commercially prepared vitamin-D-supplemented foods. The circulating concentration of calcidiol reflects skin synthesis of cholecalciferol following ultraviolet (UV) irradiation, dietary intake of calciferols, and the degree of renal tubular reclamation of calcidiol-VDBP following glomerular filtration.^10^ In dogs and cats, UV irradiation of skin results in little cholecalciferol synthesis.^a^ The circulating calcidiol-VDBP complex undergoes glomerular filtration and is delivered to the brush border of proximal tubules where it undergoes megalin-receptor-mediated endocytic reabsorption; cubulin may also be important in facilitating delivery of VDBP to the cell surface before megalin-mediated internalization. Once inside the tubular cell, the VDBP complex is degraded and calcidiol then binds to an intracellular VDBP^6^ that interacts with either 1-α-hydroxylase resulting in the synthesis of calcitriol or with 24-hydroxylase resulting in the mostly inactive metabolite 24,25(OH)_2_-vitamin D, which then undergoes side chain cleavage. Conversion of calcidiol to 24,25 (OH)_2_-vitamin D reduces the pool of calcidiol available for 1-α-hydroxylation.^11^ 24-Hydroxylase also facilitates the conversion of calcitriol to 1,24,25(OH)_3_-vitamin D, which undergoes oxidation and excretion as calcitroic acid.^11^ The expression and activity of the renal hydroxylase system is very tightly regulated; 1-α- hydroxylase and 24-hydroxylase are regulated in a reciprocal manner.^6^ 24-Hydroxylase is induced by calcitriol to decrease the effects of calcitriol and to prevent the development of hypercalcemia.^11^

Calcitriol is the naturally occurring vitamin D metabolite that has the greatest affinity for the vitamin D receptor (VDR) in tissues and circulates in picograms per milliliter. The 1-α-hydroxyl group of vitamin D metabolites is essential to provide high-affinity binding to the VDR.^10^ Calcidiol and parent vitamin D have far less avidity for the VDR. Calcidiol affinity for VDR is 100–200 times lower than that for calcitriol, but still exerts VDR activation at circulating concentrations 1000-fold higher (ng/mL) than calcitriol (pg/mL).^10^ Vitamin D receptor activation (VDRA) is the term used to indicate the effects following ligand binding of the natural vitamin D compound or analogue to the VDR. Depending on the tissue, VDRA can be upregulating or downregulating to a variety of cellular processes.

The synthetic vitamin D metabolite dihydrotachy-sterol (DHT) is intermediate in VDR affinity between that of calcitriol and calcidiol. DHT is no longer available in the USA, but is available and prescribed in other parts of the world. Some vitamin D analogues have been designed to interact with VDR to limit calcemic effects while still suppressing the synthesis of PTH.

### Renal tubular reabsorption of calcium—Effects of PTH, calcium sensing receptor, and calcitriol

Tubular reabsorption of calcium can occur across or between cells. Between 50%–60% of the filtered load of calcium is reabsorbed in the proximal tubules by passive paracellular routes; passive permeability to calcium is high in this region. Calcium reabsorption parallels that for sodium in the proximal tubules. About 15% of the filtered load is reabsorbed in the thick ascending limb of the loop of Henle by passive paracellular transport and also by active transport that is stimulated by PTH. The distal convoluted tubule and the connecting tubule account for about 10%–15% reabsorption of the filtered load by active transcellular transport. The distal nephron has very limited passive permeability to calcium in contrast to that seen in the proximal tubules.^12^–^16^ The major calcium channel expressed in the apical membrane of the renal tubule is a member of the TRP superfamily of cation channels—TRPV5 (transient receptor potential vanillinoid 5).^16^–^20^ Calcium enters the tubule through this channel is ferried across the cytosol attached to a calbindin, and is then extruded from the basolateral membrane by a Na-Ca antiporter or by a calcium cell-membrane pump into ECF. The Na-Ca exchange mechanism appears to be the most important. Entry of iCa into tubular cells leads to inactivation of this channel providing negative feedback for calcium entry. Calbindin (cytosolic calcium binding protein) expression in the intestine and distal tubule is induced by effects of calcitriol. Calbindin may provide a buffering mechanism for cytosolic calcium that does not then feed back to downregulate the expression of TRPV5, which therefore facilitates transport of more calcium across the cell. No specific extracellular ligands have been identified for TRPV5, but its action can be selectively blocked. TRPV expression is influenced by its interaction with many intracellular proteins including calmodulin.^15^,^16^,^18^,^21^,^22^ Activation of the CaR inhibits the conversion of 25-OH vitamin D to calcitriol in the proximal tubules through increase in cytosolic calcium and inhibition of 1-α-hydroxylase to ensure that circulating calcium does not become excessive. There appears to be reciprocal control mechanisms between vitamin D activation or degradation and the CaR. Calcium sensing receptor is upregulated by calcitriol.^23^ Calcitriol exerts a selective effect to increase calcium absorption in the distal tubule by increasing the expression of transport proteins (Table 1).^6^,^15^,^24^–^26^ Calcitriol knockout mice reduce expression of TRPV5, calbindin, and the Na-Ca exchanger in the distal nephron; calcitriol supplementation restores their expression.^6^ Klotho expression in the distal nephron is upregulated by calcitriol and may be involved in control of calcium reabsorption through stabilization of membrane calcium transporters.^6^,^22^

**Table 1:** Effects of calcitriol

↓ 1-α-hydroxylase activity and ↑ 24-hydroxylase– normal negative feedback loop^27^
↑ Ionized calcium (calcemic effects)^5^,^6^
↑ GI absorption calcium and phosphorus (genomic)^28^
↑ Ca pump ATPase^6^
↑ Calbindins^6^
↑ Formation of calcium channels in microvillar membranes^6^
↑ Renal resorption Calcium and Phosphorus – when blood concentration a bit low^29^,^30^
↑ Osteoclastic Bone Resorption^31^,^32^
↓ Renal Resorption Calcium – when blood calcium is too high
CaR effect – CaR in Henle's loop calcitriol induced^33^,^34^
↓ PTH Secretion – 2° ↑ Ionized Calcium)^35^
↓ PTH Synthesis – genomic inhibition^36^
Upregulation of VDR & CaR – parathyroid glands and elsewhere^23^,^37^
Parathyroid Gland Set Point Control^38^
↑ Synthesis of CaR, G proteins, calcium channels
Prevention and reversal of PTG hyperplasia -anti-proliferative effects – Counteracts effects on TGF-α and EGFR interactions induced by phosphorus^39^–^41^
↓ Renal EGFR activation —↓TACE^10^
↓ TACE (direct effect)^42^
↓ RAAS Activity^43^
↓ renin synthesis (renin gene contains VDRE)^44^
↑ FGF-23 → ↓ PTH secretion (except in advanced CKD)^45^–^47^
↑ phosphaturia & ↓ intestinal absorption (secondary to lowered calcitriol)^48^
↓ Vascular mineralization in CKD – induction of ↑ FGF-23 → phosphaturia^49^–^54^
Reversal of renal osteodystrophy from ↑ PTH^55^,^56^
Direct Renoprotective Effects – independent of ↓ PTH^10^,^57^–^59^
↓ Podocyte injury^60^
↓ Loss, hypertrophy, apoptosis^61^
Maintain slit pore membrane dynamics
↑ Nephrin^62^–^66^
↑ Podocin^62^–^66^
↓ Desmin (injury molecule)^62^–^66^
Mesangial cells – integrity preserved, ↓ proliferation
Anti-Fibrotic^67^
↓ Myofibroblast activity^68^–^70^
↓ Extracellular matrix from myofibroblasts^68^–^70^
↓ TGF-β^68^
↓ SERPINE-1^68^–^70^
↑ MMP-8 (cleaves collagen)^68^–^70^
↑ BMP7 (antagonist of TGF-β)^68^–^70^
↓EMT^68^–^70^
Antiproliferative effects (independent of PTH)-↓Glomerulosclerosis^71^
↓ RAAS Activity^72^–^74^
↓ Renin synthesis (renin gene contains VDRE)^75^
↓ ANG II generation → ↓ fibrogenic activity^75^
↓ EMT, ↓TGF-β, ↓ CTGF-β^76^
↑ Megalin expression – ↑ 25(OH)-vitamin D/VDBP uptake into proximal tubules^77^
↓ albuminuria^78^
↑ circulating 25(OH)-vitamin D^78^
↑ generation of calcitriol by providing more substrate^78^
Anti-Inflammatory^72^,^79^
↓ NF-κβ (transcription factor) & ↓TGF-β 1
↑ Uptake of 25(OH)-vitamin D at peripheral tissues –induction of members of the LDL family of receptors thought to be involved^80^
↑ Klotho – counteracts damaging effects of Ang II^49^,^50^,^54^,^81^,^82^
↓ Blood pressure^83^,^84^
95 kidney genes are controlled by calcitriol some of which provide local tissue immunologic and antiproliferative effects and many indicated above have renoprotective effects as well as cardioprotective effects.^85^

### Phosphorus regulation

Phosphate comprises about 1% of total body weight, about 85% resides in bone, 14% in cells, and 1% in serum and ECFs.^12^–^14^,^86^ Maintenance of serum phosphate within normal limits allows an optimal calcium-phosphate product for bone mineralization without deposition in soft tissues. Fine-tuning of circulating Pi is mostly under the control of renal excretion, and Pi is tightly controlled in health as there are systems independent of the classic mechanisms for calcium regulation that modulate Pi intake, utilization, and excretion. From 80%–90% of filtered Pi undergoes reabsorption in the proximal tubule by active transcellular mechanisms; any remaining Pi reabsorption occurs in the distal nephron by poorly understood mechanisms. The earliest portions of the proximal convoluted tubule have the highest density of phosphate transporters. The NaPi-IIa cotransporter is heavily localized in the brush border of the proximal tubule and accounts for about 85% of the Pi reabsorption here. The remaining 20% in this location is handled by the NaPi-IIc cotransporter.^12^–^14^,^87^–^91^

Classically, there are 4 main known regulators of phosphate metabolism: (1) dietary phosphate intake and absorption, (2) calcitriol, which can increase phosphorus resorption from bone and absorption from intestine, (3) PTH, which directly causes phosphorus resorption from bone, and indirectly activates intestinal absorption through stimulation of calcitriol production, and (4) renal tubular reabsorption of phosphorus that is stimulated by tubular filtered load of phosphorus and inhibited by PTH. However, these regulators alone cannot explain the pathophysiology of X-linked hypophosphatemic rickets (XLH) and other less well-known disorders.^92^ A group of hypophosphatemic peptides called “phosphatonins” has been identified that include matrix extracellular phosphoglycoprotein (MEPE), secreted frizzled-related protein 4 (sFRP-4), dentin matrix protein 1 (DMP1), fibroblast growth factor-7 (FGF-7), FGF-23, and Klotho. FGF-23 was first identified in 2000 as a major regulator of phosphorus^93^ with other factors such as MEPE and DMP-1 that are now thought to mostly act by modulating activity of FGF-23.^94^,^95^

### FGF-23 and Klotho

FGF-23 is a 251-amino-acid protein that is synthesized and secreted by bone cells, mainly osteocytes, but also osteoblasts.^96^ The overall effect of FGF-23 is to decrease serum Pi by increasing its renal excretion, and decreasing intestinal absorption of Pi via diminished calcitriol. The half-life of intact FGF-23 is about 58 minutes in healthy human subjects.^97^ The N-terminal peptide binds to tissue receptors, and the C-terminal binds to Klotho. Receptors for FGF-23 are present in many tissues, but only the kidneys and PTGs can respond to FGF-23 as they have both receptor and Klotho. FGF-23 activity requires the co-factor Klotho, which is a transmembrane protein. Klotho is an obligate co-receptor, and is required for FGF-23 to interact with its receptor. The kidney is the major source of Klotho, but Klotho expression is also found in brain, heart, PTG, testis, aorta, colon, pituitary gland, thyroid gland, and pancreas. In the kidney, Klotho is found primarily in the distal convoluted tubules, but also to a lesser extent in the proximal convoluted tubules. A bioactive fragment of “tethered” Klotho is present in blood, cerebrospinal fluid, and urine.^98^

Animals with Klotho deficiency show signs of FGF-23 deficiency with high serum phosphate and calcitriol concentration.^99^ Transgenic mice that overexpress Klotho live longer than control mice;^100^ this has also been seen in people confirming Klotho as an antiaging factor.^86^,^101^ Mice with the negative mutated Klotho gene show hyperphosphatemia, increased serum calcitriol concentrations, development of extraskeletal calcification, rapid aging, and early death.^100^–^102^ Klotho may also have phosphate regulatory effects independent of FGF-23.^103^

FGF-23 suppresses the expression of apical membrane NaPi-IIa and IIc cotransporters, which mediate the phosphate reabsorption by the kidney proximal tubules.^104^ Phosphorus exits the basolateral tubular cell membrane by a transporter yet to be characterized. FGF-23 thus causes a decrease in phosphate reabsorption, leading to phosphaturia and hypophosphatemia.^86^ PTH also stimulates phosphaturia; PTH reduces Pi reabsorption (phosphaturia) by favoring endocytic removal of these transporters from the brush border, their internalization, and lysosomal degradation. Megalin is important in regulating the response of the NaPi-IIa transporter to PTH that results in phosphaturia and calcitriol upregulates megalin expression in the kidney.^10^ The effects of PTH are immediate and the effects of FGF-23 take more time. Calcitriol has an opposing effect by increasing expression of renal NaPi-IIc cotransporters and tubular reabsorption of Pi.^10^ The effects of FGF-23 on tubular phosphate reabsorption are independent of PTH and calcitriol. FGF-23 inhibits 1-α-hydroxylase in the kidney and stimulates 24-hydroxylase activity, thereby decreasing calcitriol synthesis and increasing calcitriol metabolism to the inactive 1,24,25(OH)_3_-vitamin D.^105^ In addition, FGF-23 inhibits the secretion of PTH before uremia is advanced, but this is a minor effect as the major regulator of PTH secretion is serum iCa.

Dietary phosphorus intake results in increased secretion of FGF-23 causing increased phosphaturia and decreased calcitriol production. Decreased calcitriol concentration result in decreased intestinal absorption of both phosphorus and calcium. The dominant effect of FGF-23 is to maintain phosphorus homeostasis. Calcitriol increases enterocyte phosphate transport into the circulation through increased expression of the NaPi-IIb cotransporter;^6^ high concentration of calcitriol stimulate FGF-23 production creating a feedback loop as FGF-23 decreases the production of calcitriol. Acute effects of vitamin D are to increase proximal tubular reabsorption of Pi through increased expression of the NaPi-IIa cotransporter; chronic vitamin D exposure via FGF-23 production leads to decreased Pi absorption due to decreased expression of these transporters. With dietary restriction of phosphorus, there will be lower concentration of FGF-23.

An increase in serum phosphorus results in an increase in expression of FGF-23. Increased FGF-23 results in an inhibition of 1-α-hydroxylase activity and an increase in 24-hydroxylase activity in the kidney, both of which decrease circulating concentration of calcitriol. Decreased circulating calcitriol decreases phosphorus absorption in the intestine, contributing to the decrease in serum phosphorus. Increased FGF-23 indirectly decreases the NaPi-IIb cotransporter in the intestine following reduced synthesis of calcitriol that helps to decrease gastrointestinal (GI) absorption of phosphorus. Increased FGF-23 increases fibroblast growth receptor (FGF-R)/Klotho gene expression in the kidney, which decreases the NaPi-IIa cotransporter in the kidney, decreasing tubular reabsorption of phosphorus, thereby lowering serum phosphorus. Increased FGF-23 also increases FGF-R/Klotho in the PTG and decreasing PTH synthesis. This also indirectly lowers serum phosphorus through the decreased production of calcitriol.

## Renal Secondary HPTH

### Development of secondary HPTH

**Figure 2:**
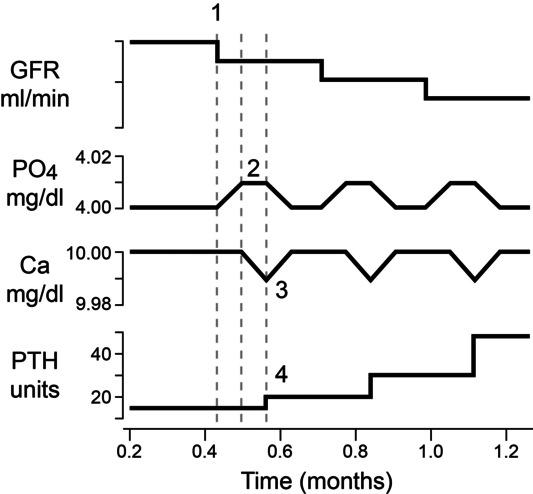
Development of renal secondary hyperpara-thyroidism–classic theory. (Adapted from Chew DJ, DiBartola SP, Schenck PA. Canine and Feline Nephrology and Urology, Elsevier 2010.)

Renal secondary HPTH occurs when PTH synthesis and secretion become excessive during kidney disease and is the result of increased secretion of PTH by each chief cell as well as the increased number of chief cells due to PTG hyperplasia. Untreated secondary HPTH leads to altered bone histology, bone fragility, skeletal deformities, growth retardation, and cardiovascular calcifications in human patients.^2^,^3^,^106^ iCa is either normal or low in secondary HPTH. PTG hyperplasia is the predominant cause of increased PTH secretion.^107^,^108^ Classic theory for the development of renal secondary HPTH is described in the original “trade-off” hypothesis (Figure 2). Step 1 is the loss of nephron mass by some chronic disease process. With every loss of nephron mass, there is a small increase in circulating phosphorus (maximal increase achieved at step 2). iCa decreases (maximal decrement achieved at step 3) as a consequence of the increase in the serum phosphorus. PTH increases (step 4) in response to the decreased iCa. This theory ignores the important genomic effects of calcitriol required to inhibit PTH synthesis.

**Figure 3:**
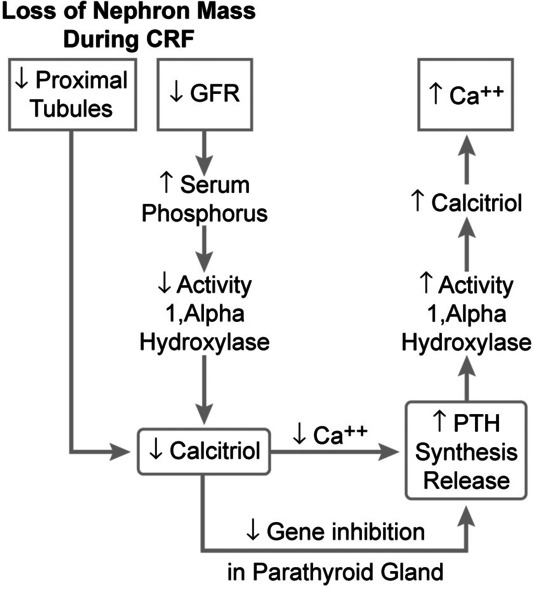
Development of renal secondary hyperpara-thyroidism–calcitriol trade-off hypothesis. (Adapted from Chew DJ, DiBartola SP, Schenck PA. Canine and Feline Nephrology and Urology, Elsevier 2010.)

**Figure 4:**
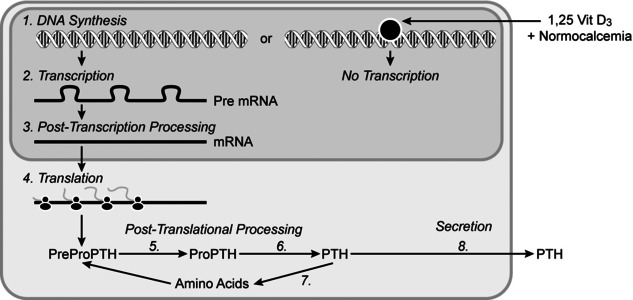
Calcitriol's effect to genomically control the synthesis of parathyroid hormone. (Adapted from Chew DJ, DiBartola SP, Schenck PA. Canine and Feline Nephrology and Urology, Elsevier 2010.)

Calcitriol deficit is an important factor leading to the uncontrolled synthesis and secretion of PTH. Adequate amounts of calcitriol and iCa inside the PTG are required to inhibit gene transcription in the pathway for PTH synthesis.^4^ In kidney disease, there are fewer healthy proximal tubule cells containing the mitochondrial 1-α-hydroxylase enzyme system necessary to form calcitriol from precursor calcidiol. Decreased calcitriol decreases intestinal calcium absorption leading to hypocalcemia; as iCa concentration falls, the secretion of PTH is stimulated. The increased PTH concentration can restore calcitriol and iCa in early stages of CKD when enough proximal tubular cells remain that are capable of calcitriol synthesis. The “calcitriol trade-off” hypothesis was developed based on concepts explained in Figure 3. Chronic kidneys diseases result in the loss of tubular mass. Since calcitriol is synthesized within the renal tubules, deficits of calcitriol synthesis occur. Increases in phosphorus burden within the body also contribute to decreased calcitriol as activity of the 1-α-hydroxyalse system within the renal tubules is impaired. Circulating calcitriol provides an important negative effect on PTH synthesis by inhibition of gene transcription within the PTG cell nucleus. The combination of low calcitriol and low iCa allows high concentration of PTH to be synthesized and secreted. The higher concentration of PTH upregulates the activity of the 1-α-hydroxylase system within the renal tubules, returning calcitriol production to normal (if there is a sufficient residual renal tubular mass). The restored calcitriol concentrations in the circulation are maintained at the expense of a higher than normal PTH. There is a negative impact from chronically high PTH concentration on a variety of organs including the kidneys. Calcitriol exerts its genomic effect within the parathyroid cell nucleus to inhibit transcription of the gene, which then decreases PTH synthesis and secretion (Figure 4). Basal condition in the PTG is to synthesize PTH unless adequate calcitriol and iCa are present. Calcitriol in concert with iCa and other transcription factors serve as an “off” switch by binding to a silencing region of the DNA.

Traditionally, secondary HPTH was thought to be primarily the result of a loss of renal function with a direct reduction of 1-α-hydroxylase, with a decrease in calcitriol production and oversecretion of PTH. However, with a reduction in tubular mass, both 1-α-hydroxylase and erythropoietin secretion should be decreased, and evidence shows that erythropoietin secretion remains unaffected in earlier stages of CKD when decreased calcitriol is present.^105^ Through the actions of FGF-23, serum phosphorus concentration is maintained at the expense of calcitriol, and the decrease in calcitriol is greater than would be predicted from the numbers of nephrons lost. The early FGF-23-mediated decrease in calcitriol facilitates PTH secretion, initiates secondary HPTH,^105^ and can occur prior to the development of phosphaturia.^109^

In human CKD, FGF-23 concentrations increase gradually before an increase in serum phosphorus. In people, there is a significant correlation between serum FGF-23 concentration and glomerular filtration rate (GFR).^104^ Since FGF-23 increases early in CKD, it may be a better indicator of early kidney disease than other clinical parameters. It was originally thought that the increase in FGF-23 in CKD occurred primarily as a result of decreased renal clearance. It is now thought that although FGF-23 secretion is increased in CKD, there is end-organ resistance to FGF-23 due to a deficiency of the Klotho cofactor. Klotho mRNA expression is decreased in the PTG of CKD patients.^45^ As a result of a downregulation of the Klotho/FGF-R complex in the PTGs, an increase of circulating FGF-23 does not decrease PTH concentration in CKD.^45^

**Figure 5:**
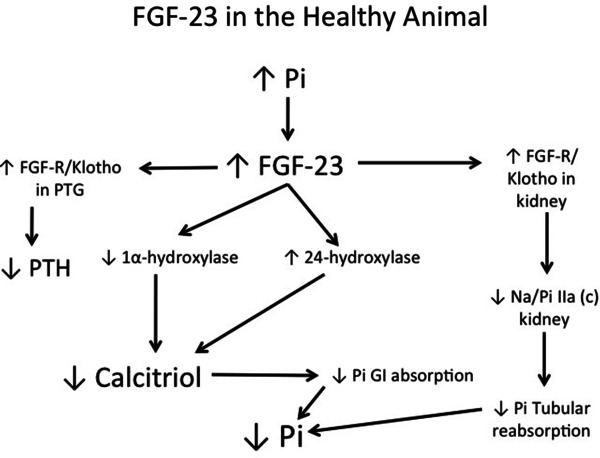
The role of fibroblast growth factor-23 (FGF-23) in the healthy animal. An increase in serum phosphorus results in an increase in expression of FGF-23. (Adapted from Schenck PA: Pathogenesis of Secondary Hyperparathyroidism–ACVIM Forum, Anaheim, California 2010.)

**Figure 6:**
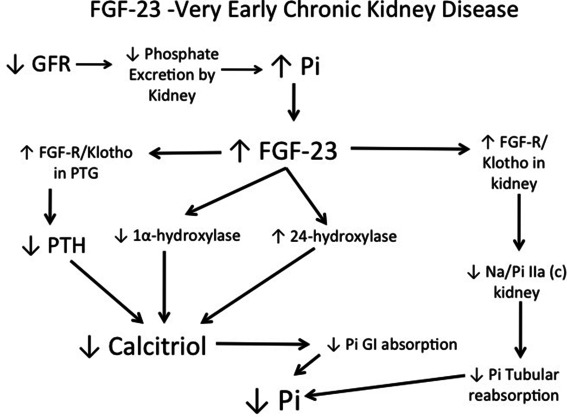
The role of FGF-23 in very early chronic kidney disease. Decreasing glomerular filtration rate (GFR) decreases the phosphate excretion by the kidney, resulting in an increase in serum phosphorus concentration. (Adapted from Schenck PA: Pathogenesis of Secondary Hyperparathyroidism–ACVIM Forum, Anaheim, California 2010.)

Figure 5 shows the FGF-23 response in a healthy animal following an increase in circulating phosphorus. Figure 6 shows the role of FGF-23 in very early CKD. Decreasing nephrons and GFR decrease the phosphate excretion by the kidney, resulting in an increase in serum phosphorus concentration. This increase in serum phosphorus stimulates production of FGF-23. Normal physiologic events occur as shown in the Figure 5, and serum phosphorus decreases in response to the increase in FGF-23. Thus, FGF-23 increases urinary phosphate excretion and indirectly decreases gastrointestinal Pi absorption via decreased calcitriol synthesis. These effects allow serum phosphorus to be maintained within a “normal” range until CKD becomes more advanced. Increased circulating FGF-23 is most likely responsible for the maintenance of normal serum phosphorus concentration in very early CKD.^105^

**Figure 7:**
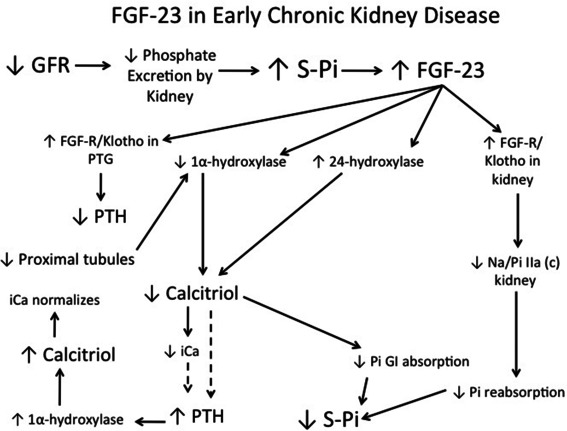
The role of FGF-23 in early chronic kidney disease. As the GFR further declines, serum phosphorus elevation becomes more severe with an increase in FGF-23 production. (Adapted from Schenck PA. Pathogenesis of Secondary Hyperparathyroidism–ACVIM Forum, Anaheim, California 2010.)

Figure 7 shows the role of FGF-23 in early CKD. As the GFR further declines, serum phosphorus increases becomes more severe with an increase in FGF-23 production. Basic physiologic events occur as described in Figure 5. With decreasing kidney function, the absolute number of proximal tubules decreases, contributing to a decreasing activity of 1-α-hydroxylase, thereby causing a further decrease in calcitriol production. With this degree of calcitriol decrease, the iCa concentration begins to drop which stimulates PTH secretion (secondary HPTH). The increase in PTH concentration is able to upregulate the activity of 1-α-hydroxylase in functional kidney tubules, causing an increase in calcitriol concentration. The increase in calcitriol production will help to normalize the iCa concentration, but then contributes to increasing serum phosphorus. Thus in this stage of kidney disease, a normal iCa concentration with increased PTH concentration and normal-to-high serum phosphorus concentration may be observed.

The upregulation of FGF-23 in CKD results in early calcitriol deficiency, which initiates secondary HPTH. Secondary HPTH is typically the first metabolic complication noted in human patients with CKD, and occurs prior to hyperphosphatemia. In cats and dogs with early CKD, there is no correlation between serum phosphorus concentration and PTH concentration; a number of dogs and cats with secondary HPTH have normal serum phosphorus concentrations.^110^,^111^

**Figure 8:**
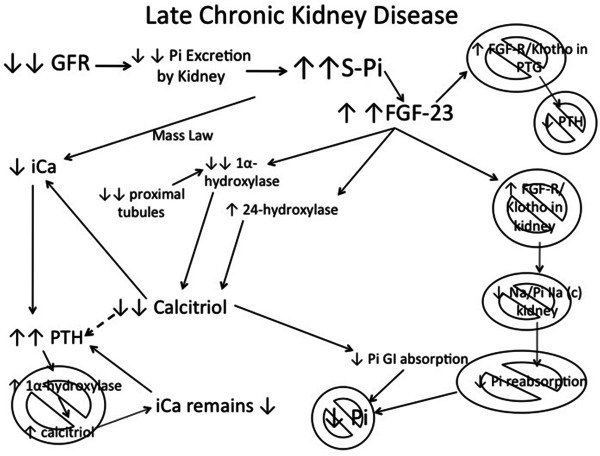
The role of FGF-23 in late chronic kidney disease. As more kidney function is lost, there is an absolute decrease in GFR, leading to more significant elevations of serum phosphorus concentration. (Adapted from Schenck PA: Pathogenesis of Secondary Hyperparathyroidism–ACVIM Forum, Anaheim, California 2010.)

Figure 8 shows the role of FGF-23 in late CKD. As more kidney function is lost, there is an absolute decrease in GFR, leading to more significant increases in serum phosphorus concentration. This in turn leads to increasing FGF-23 concentrations. As a result of both mass law effects and the absolute decrease of calcitriol production due to loss of functional proximal tubules, iCa concentration decreases. The low iCa concentration stimulates PTH production, but the increased PTH is unable to upregulate calcitriol synthesis due to a lack of functional tubules. Thus, the iCa concentration remains low with a continual increase in PTH secretion in an attempt to normalize iCa concentration. With the absolute decrease in renal tubules, there is decreased Klotho in the kidney and PTG, with end-organ resistance to the actions of FGF-23. Consequently, FGF-23 actions to excrete phosphorus or blunt PTH synthesis are minimized. In addition, the upregulation of 24-hydroxylase increases the degradation of any remaining calcitriol. Thus in late CKD, the iCa concentration is typically low, with increased serum phosphorus concentration and a significant secondary HPTH develops.

In several human studies, serum FGF-23 concentration was a better outcome predictor than serum phosphorus concentration in CKD. Increased FGF-23 concentrations have been independently associated with faster progression of CKD, and treatment-resistant secondary HPTH.^112^ Higher concentration of serum phosphorus (even if within the reference interval) and FGF-23 have been associated with increased mortality in all stages of CKD.^113^ This is most likely related to increased vascular calcification and left ventricular hypertrophy as a result of increased FGF-23.^114^ However, there have been no studies yet in dogs to evaluate circulating FGF-23 concentrations in CKD. FGF-23 has been measured in cats with CKD and hyperthyroidism and is discussed later.

At this time, there are no drugs available that directly lower FGF-23 concentrations. However, serum FGF-23 concentration can be indirectly lowered by the use of oral intestinal phosphate binders. In a recent study, people with stage 4 CKD were treated with either calcium acetate or sevelamer (phosphate binders) for 8 weeks.^115^ Both calcium acetate and sevelamer were able to decrease serum phosphorus in these patients; this effect was more pronounced in the sevelamer-treated group. Both treatments resulted in a decrease in FGF-23; this decrease was more pronounced in the sevelamer-treated group. There have been no studies in dogs or cats to evaluate vascular disease and FGF-23 in CKD.

Tertiary HPTH refers to the condition of a subset of patients with CKD who develop ionized hypercalcemia and excessive PTH secretion that is not inhibited by high serum iCa concentration.^116^–^119^ It is likely that such patients had high PTH concentrations in association with normal or low serum iCa (renal secondary HPTH) earlier in the clinical course of CKD.^4^ The set point for control of PTH secretion may be altered in CKD such that higher concentrations of iCa are necessary to inhibit PTH secretion. Decreased serum calcitriol concentrations, decreased numbers of calcitriol receptors in the PTG, and decreased calcitriol–VDR interactions with chief cell DNA caused by uremic toxins^120^ may contribute to this increase in set point. The calcium receptor establishes the set point and depends on calcitriol for synthesis of its mRNA from the parathyroid cells’ DNA. Tertiary HPTH is an example of a condition associated with set point change and ionized hypercalcemia that benefits from treatment with calcitriol due to its induction of the calcium receptor.^23^

### Experimental CKD in dogs and cats–PTH, calcidiol, calcitriol, and FGF-23

Early studies in experimental dogs demonstrated progressive increases in PTH to as much as 20-fold over baseline following renal mass reduction. Increases in PTH were demonstrated even during early stages of nephron reduction (1/3, 2/3, 5/6 & 11/12 partial nephrectomy).^121^ A parathyroidectomy/nephrectomy model was also studied to see its effects on survival and renal function of dogs with induced CKD.^122^ The parathyroidectomy group had lower mortality and greater stabilization of GFR that approached statistical significance (*P* < 0.06) between 12 and 24 months, better-preserved bone mineralization, and less severe soft tissue mineralization. GFR was stable in most dogs with parathyroidectomy between 12 and 24 months, whereas most dogs without parathyroidectomy experienced progressive decrease in GFR.^122^ Increases in PTH were prevented when dietary intake of phosphorus was substantially reduced (100 mg/day from 1200 mg/day) even during advanced stages of CKD.^123^ This was a pivotal study in determining that phosphorus control systems were important in the genesis of renal secondary HPTH. Further studies in dogs confirmed that reduction in dietary phosphorus intake proportional to the reduction in GFR prevented increases in PTH.^121^ In a later study in dogs with 15/16th nephrectomy, the influence of dietary phosphorus and protein intake on survival and PTH concentration were studied over 24 months.^124^ Survival was significantly longer in dogs consuming the lower phosphorus diet, but not influenced by dietary protein intake.^124^ PTH concentration was significantly lower in the dogs consuming low dietary phosphorus.^124^

In another study in dogs with experimental reduction in nephron mass, dietary phosphorus restriction was associated with a reduction of PTH by approximately 70%.^125^ This reduction was observed in the absence of a concomitant increase in concentration of iCa or calcitriol.^125^ These results support that whenever nephron losses occur and GFR diminishes, phosphate excretion must decrease transiently until increased PTH enhances phosphaturia by decreasing tubular reabsorption of Pi. It is important to note that even though the total phosphorus excretion decreases once the total GFR is below 25%–30% of normal, the phosphorus excretion per nephron is greater than normal and this is partially maintained by increased PTH concentration.^126^ Hyperphosphatemia can induce tissue mineralization, especially in the kidneys leading to progression of the renal insufficiency.^127^ Results from these studies provided support to the trade-off hypothesis in which preservation of phosphorus and calcium homeostasis is maintained at the expense of increased circulating concentration of PTH.^128^,^129^ This “trade-off” concept^130^ has now been extended to include changes in calcitriol^131^ and FGF-23.^48^

A high parenteral dose of cholecalciferol or calcidiol (1.25 μg) given to uremic rats produced no effect on intestinal calcium transport due to their inability to convert these compounds into calcitriol-vitamin D.^132^ In a study in dogs, oral doses of cholecalciferol (100 μg every other day for 2 weeks followed by 50 μg every other day for the next 2 weeks) to uremic or anephric dogs resulted in a 4-fold increase in calcidiol and a 1-fold increase in the concentration of circulating calcitriol.^133^ This increase in calcitriol was attributed to extrarenal genesis, which is now well established to occur. Calcidiol administration to dogs with moderate renal failure for 2 weeks did not affect circulating calcitriol concentration, but doubled calcitriol production in dogs with severe renal insufficiency.^134^ These data suggest that the basal concentration of calcitriol affects the synthesis of calcitriol in response to calcidiol administration in uremic dogs.^134^

In a landmark study, the chronic interaction between vitamin D status and dietary phosphorus intake in CKD was studied over 2 years in 3 groups of dogs with 5 of 6 nephrectomy.^135^ Group 1 had no dietary phosphate restriction, Group 2 had proportional reduction of dietary phosphate to match their decrease in GFR, and Group 3 had dietary reduction of phosphate intake and supplementation with 25(OH)-vitamin D_3_. PTH progressively increased in Group 1 dogs throughout the 2 years, Group 2 dogs had normal PTH concentration for the first year and modest increases during the second year, and Group 3 dogs did not increase their PTH concentrations throughout the 2 years and had better bone histopathology scores, but they were hypercalcemic due to overdosage of calcidiol.^135^ Reversal of established renal secondary HPTH occurred in 7 chronically uremic dogs following proportional reduction of dietary intake of phosphorus; PTH returned to within or close to the normal range.^136^ This work in dogs provided strong evidence that vitamin D metabolites in concert with dietary phosphorus restriction could be useful in the prevention and reversal of renal secondary HPTH associated with CKD. Phosphorus restriction reverses secondary HPTH as long as the kidneys are able to convert inactive vitamin D into active vitamin D maintaining calcium homeostasis.^137^,^138^ Otherwise, supplementation of activated vitamin D compounds is necessary to control secondary HPTH.^139^

The effect on survival and on low PTH concentrations compared to normal dietary phosphorus intake (0.42% or 1.56% dry matter) was reported in the earliest study in cats with surgically induced CKD.^140^ Survival time did not differ between treatment groups though this could be accounted for by the short follow-up time (65–343 days) and the slowly progressive nature of CKD in cats.^140^ Renal histopathology was however remarkably different between dietary treatment groups. Cats fed with a normal phosphorus-containing diet had far more severe mineralization, fibrosis, and infiltration of mononuclear cells into their kidneys. Circulating PTH was 30- to 70-fold higher than that in healthy cats for CKD cats eating a diet not restricted in phosphorus. Cats with CKD eating a diet restricted in phosphorus had a minimal PTH increase above that for healthy cats.^140^ PTH was measured using a carboxyterminus assay that detected intact PTH as well as a large amount of inactive carboxy-fragments of PTH that accumulate during CKD due to failed urinary elimination. Preservation of relatively normal renal histopathology in cats eating the diet restricted in phosphorus could be attributed to the degree of decrement in circulating PTH and phosphorus that was achieved. Vitamin D metabolites were not measured in this study.

In one experimental study, 3 daily IV doses of calcitriol (400 ng) were not effective in reversing HPTH in uremic dogs when PTH was measured 3 days after the first treatment.^137^ In another study, oral calcitriol dosed at 30–50 ng/kg/day for 14 weeks in dogs with induced renal failure led to significantly decreased appetite and dramatic weight loss despite the lack of hypercalcemia.^141^ This dose is much higher than what is used clinically today in human and veterinary medicine. The absence of hypercalcemia was likely caused by severe anorexia and consequent lack of calcium for intestinal absorption. Calcitriol concentrations increased during treatment, but PTH was not measured.^141^ In a later study in dogs with experimentally induced renal secondary HPTH, an oral calcitriol dosage of 6.6 ng/kg/day resulted in suppression of PTH secretion within 30 days in most dogs, but hypercalcemia occasionally developed at this dose.^142^

A 4X decrease in 1,25-(OH)_2_-VDR density has been reported in the PTGs of experimentally uremic dogs.^143^ The VDR in the PTG is upregulated by calcitriol; so, it is likely that low circulating calcitriol during CKD accounts for most of the downregulation of the VDR in this tissue. This contributes to decreased inhibitory action of calcitriol on the synthesis and secretion of PTH (less VDR–ligand interaction), and is important in the development of HPTH associated with CKD.^143^

### Naturally occurring CKD in dogs and cats—PTH, calcidiol, calcitriol, and FGF-23

Renal secondary HPTH is commonly documented in dogs and cats with spontaneous CKD. Overall frequency of renal secondary HPTH was 76% in a recent study in dogs with CKD, encountered in 36% of International Renal Interest Society (IRIS) stage 1, 50% in stage 2, 96% in stage 3 and 100% in IRIS stage 4.^110^ An increasing frequency of renal secondary HPTH was similarly found in cats with CKD,^144^ affecting 84% of cats overall including 47% of cats with stable azotemia without clinical signs, to 100% of cats with decompensated CKD. Hyperphosphatemia is commonly found in CKD patients with secondary HPTH, but secondary HPTH can be encountered in both dogs and cats with serum phosphorus within the reference interval. Hyperphosphatemia was noted in 18%, and secondary HPTH in 36% of dogs in IRIS stage 1 dogs.^110^ The concept that renal secondary HPTH can precede development of hyperphosphatemia in CKD has not been well appreciated in veterinary medicine. Serum phosphorus in the upper reference interval has recently been associated with increased PTH in CKD dogs^110^ confirming an earlier report of this association.^145^

The feeding of a renal diet compared to a maintenance diet to azotemic dogs with naturally ocurring CKD (IRIS stage 3) resulted in increased survival and preservation of renal function.^146^ PTH was increased at baseline and throughout the study, but was not significantly different between feeding groups at baseline or after 24 months of feeding. Serum phosphorus was not significantly different between treatment groups, and dogs in both groups received intestinal phosphorus binders.^146^ Similar results for survival and preservation of renal function (based on serum creatinine concentration) were obtained in cats with IRIS stage 2 and 3 spontaneous CKD treated with a renal or maintenance diet.^147^ PTH concentrations were increased at baseline in both treatment groups and did not differ throughout the 24 months of this study. Vitamin D metabolites were not measured in either the dog or cat study. PTH control was not achieved despite dietary phosphorus restriction and use of intestinal phosphate binders, so survival benefit appeared independent of PTH in these 2 studies.

Similarly, in a different prospective study in cats with naturally occurring CKD, dietary phosphorus restriction in conjunction with the use of phosphorus binders when needed, led to a significant increase in survival time (633 versus 264 days).^148^ In contrast to the previous study,^147^ cats fed a phosphorus-restricted diet had significantly lower phosphorus (5.7 versus 6.3 mg/dL) and plasma PTH concentrations (86 versus 216 pg/mL) at mid-survival time point.^148^

PTH control was seen in an early study in cats with naturally occurring CKD treated by feeding a veterinary renal diet restricted in protein and phosphorus.^111^ A dramatic reduction of circulating PTH was documented in cats eating a renal diet compared to a maintenance diet. PTH declined by an average of 70% from baseline in cats fed a phosphorus-restricted diet whereas PTH continued to increase from baseline in those cats eating maintenance foods. Decreased circulating PTH usually occurred within the first 30–50 days of feeding the renal diet. Plasma phosphate concentrations decreased in cats consuming the renal diet whereas increased phosphate concentrations developed for cats eating the maintenance diet. Calcitriol was measured, but calcidiol was not measured in these cats. Calcitriol was measured in 13 cats receiving the renal diet and was normal at baseline and did not change over 4–7 weeks.^111^ In one large study, calcitriol concentration progressively decreased with increasing severity of azotemia at a time of initial diagnosis and reached statistical significance in cats characterized as uremic (mean serum creatinine: 316 μmol/L; 4.1 mg/dL) and in end-stage cats (mean serum creatinine 909 μmol/L; 11.9 mg/dL).^144^ Calcitriol concentrations were within the reference interval in compensated CKD cats (mean serum creatinine 229 μmol/L [3.0 mg/dL]). PTH progressively increased with increasing severity of azotemia in the same study by approximately 1.6-, 4.7-, and 13.7-fold above the upper limit of the reference interval for compensated, uremic, and endstage CKD cats, respectively.^144^

Dogs with naturally occurring CKD (creatinine: >159.1 μmol/L [>1.8 mg/dL]; mean: 203.3 ± 71 μmol/L [2.3 ± 0.8 mg/dL]) were treated with either a maintenance diet or a renal diet for 8 weeks in a crossover design. PTH concentrations were increased approximately 3-fold over healthy control dogs at the conclusion of this study, but were not different from each other by feeding group. While on the low protein and phosphorus renal diet, dogs gained weight in comparison to the maintenance diet dogs, which lost weight. Calcitriol concentrations at the end of the study were not different by feeding group or healthy controls.^149^

Oral calcitriol treatment increased survival in a placebo-controlled randomized study in 37 azotemic dogs with CKD for 1 year.^b^ Dogs of both groups were also fed a commercial therapeutic renal diet. The mean initial serum creatinine was 353.6 μmol/L [4.0 mg/dL] with a range of 176.8–556.9 μmol/L [2.0– 6.3 mg/dL] at the time of enrollment. Calcitriol was initiated at 2.5 ng/kg/day and then adjusted to 0.75–5.0 ng/kg/day according to serial determinations of iCa and PTH. Median survival time was 365 days for the calcitriol treatment group and 250 days for the placebo-treated group. The all-cause mortality rate at 1 year was 63% in the placebo-treated dogs compared to 28% in the calcitriol-treated dogs. Results from this study in CKD dogs is similar to that seen during calcitriol treatment in human patients with CKD prior to dialysis.^150^,^151^ Increased survival could in part be attributed to the targeted concentration of PTH achieved as well as VDRA achieved by calcitriol treatment that provides renoprotective effects independent of lowering PTH. Increased survival may also have been derived from benefits of calcitriol achieved in peripheral tissues. Increased life span was associated with a decreased rate for the progression of CKD in these dogs.^152^ A similar study in cats with CKD failed to show benefit of calcitriol treatment over placebo for 1 year. Results of this study are difficult to interpret due to the short duration of treatment period in relation to the inherently more slowly progressive nature of CKD in cats compared to that in dogs.^152^

Ten healthy and 10 cats with CKD (mean ± SD creatinine: 249.3 ± 45.1 μmol/L [2.82 ±0.51 mg/dL]) were treated for 14 days with calcitriol 2.5 ng/kg/day and later (after 7-day washout period) with 8.75 ng/kg every 84 hours. Even though baseline PTH differed among groups, PTH did not significantly decrease after calcitriol treatment in either group or treatment interval.^153^ Calcitriol concentration did not change regardless of treatment group or interval. The most likely reasons for lack of effect in these cats may be due to low calcitriol dose, lack of longer term follow-up, and that PTH was not significantly increased prior to treatment. Increased PTH is expected in cats with azotemic CKD based on previous studies. However, PTH was measured using a different assay in the past.^144^

**Figure 9:**
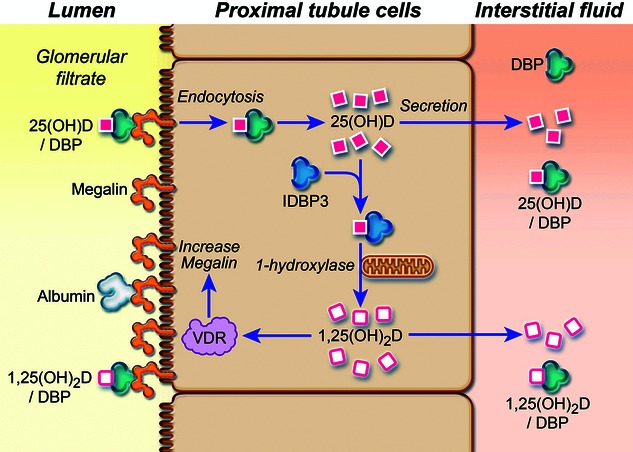
Megalin-mediated tubular recovery of vitamin D metabolites following glomerular filtration. (Adapted from: Dusso A, Gonzalez EA, Martin KJ. Vitamin D in chronic kidney disease. Best Pract Res Clin Endocrinol Metab 2011; 25: 647–655, and Dusso A, Arcidiacono MV, Yang J, Tokumoto M. Vitamin D inhibition of TACE and prevention of renal osteodystrophy and cardiovascular mortality. J Steroid Biochem Mol Biol 2010; 121: 193–198.)

Mean concentrations of calcitriol and calcidiol in dogs with both stable and unstable naturally occurring CKD were significantly lower than healthy controls; though concentrations were often still within the reference interval,^154^ the reported reference interval in this study was wide. In a different study in dogs, mean calcitriol concentrations were lower in the CKD group compared to healthy dogs even at early stages.^145^ The decrement of calcidiol was considerably more prominent than the decrease in calcitriol.^154^ It is not surprising that calcitriol was not suppressed below the reference interval due to the correcting effects explained by the calcitriol trade-off hypothesis in which increased PTH promotes more renal synthesis of calcitriol. The decreased concentration of circulating 25(OH)_2_-vitamin D was attributed to lack of dietary intake of vitamin D and to increased urinary losses;^154^ increased urinary losses of the complex of calcidiol with VDBP may be explained by the lack of megalin expression in renal tubules during CKD (Figure 9).^155^ Circulating calcidiol and calcitriol are largely bound to VDBP. This complex passes freely into tubular filtrate. The D-metabolite/VDBP complex is recovered from tubular filtrate following binding to megalin along the proximal tubules. Inside the tubular cell, the D metabolite dissociates from the VDBP and either re-enters the circulation or is acted upon by mitochondrial hydroxylases to be activated to calcitriol or catabolized to 24,25(OH)_2_-vitamin D. Vitamin D metabolites that exit the tubular cell bind again to VDBP upon reentry into the circulation. This is an example of classic receptor-mediated endocytosis and is important in maintaining circulating calcidiol concentration. Tubular reabsorption of albumin is importantly mediated following binding to megalin also. Megalin expression is reduced in CKD due to loss of renal mass and deficit of calcitriol. In CKD, megalin expression is decreased resulting in albuminuria and loss of vitamin D metabolites along with VDBP into urine.

The circulating concentration of calcidiol was significantly decreased in 19 dogs with naturally occurring CKD compared to healthy control dogs.^156^ Twenty-six percent of the CKD dogs were categorized as IRIS stage 2, 16% as stage 3, and 58% as stage 4. Control dogs consumed a variety of nonspecified commercially available or home-cooked diets, as did most of the CKD dogs (3 dogs were fed a renal diet alone or combined with other foods). Circulating calcidiol concentrations are importantly related to calciferol intake in the diet of dogs.^157^ Diet history indicated that food intake was markedly reduced in over half of the CKD dogs. Control dogs had a median calcidiol concentration of 36 ng/mL (94 nmol/L) with a range of 20–105 ng/mL (50–262 nmol/L). Calcidiol concentrations in the 25%–75% of healthy dogs were approximately from 30 to 50 ng/mL (75–125 nmol/L). CKD dogs had a median calcidiol value of 14.5 ng/mL (36 nmol/L) with a range of 3–54 ng/mL (36–134 nmol/L). Calcidiol concentrations in the 25th–75th percentile of CKD dogs were approximately from 10 to 30 ng/mL (25–75 nmol/L). The concentration of calcidiol did not vary by severity of CKD as categorized by IRIS staging. Calcidiol concentrations were positively correlated to serum albumin and inversely proportional to the magnitude of proteinuria in the CKD dogs. Low concentration of circulating calcidiol was attributed to decreased dietary intake of cholecalciferol associated with anorexia and vomiting and increased loss of calcidiol/VDBP complex into urine in those with proteinuria. We have anecdotally observed some CKD dogs in which the circulating calcidiol concentration was low despite adequate intake of food supplemented with parent vitamin D. Circulating calcitriol was not measured in this study.^156^

In one study, calcitriol concentration in dogs with naturally occurring disease progressively decreased with the severity of the CKD. Calcitriol concentration was statistically different in dogs in IRIS Stage 3 when compared to other IRS stages of CKD.^110^ Cats with secondary renal HPTH tend to maintain normal iCa concentration and calcitriol concentration (80% of the time) until end-stage CKD (blood urea nitrogen (BUN) > 140 mg/dL).^144^ Dogs behave in the same way where iCa is statistically significantly lower only in IRIS stage 4 and calcitriol in stages 3 and 4.^110^ A serum phosphorus of 4.5–5.5 mg/dL is approximately 85% sensitive and specific in predicting secondary renal HPTH.^110^ The development of guidelines aiming to maintain phosphorus concentration below 4.5 mg/dL is based on the fact that PTH concentration are frequently increased in CKD dogs with upper normal reference interval serum phosphorus values (4.5–5.5 mg/dL). Increased PTH occurs in many instances prior to hyperphosphatemia.^110^,^145^ This phenomenon is also documented in people; it is thought that serum phosphorus is an insensitive biomarker of phosphorus dysregulation and increases in serum phosphorus above the so-called “normal range” are a late finding in CKD.^113^

### FGF-23 measured in blood

Circulating FGF-23 concentrations have been reported in cats, but not dogs. FGF-23 was measured in ethylenediamine tetra-acetic acid (EDTA) plasma using a human intact FGF-23 ELISA assay, which was validated for use in cats.^c^ FGF-23 concentrations of azotemic (creatinine: ≥ 176.8 μmol/L [≥2.0 mg/dL]) and nonazotemic (creatinine: ≤141.4 μmol/L [≤1.6 mg/dL]) geriatric cats (>9 years) were measured. These cats were subdivided into separate groups depending on their level of azotemia and serum phosphorus concentration according to the IRIS staging system. FGF-23 concentrations were significantly higher in more azotemic and hyperphosphatemic cats. FGF-23 concentrations in azotemic cats were higher as serum phosphorus concentrations increased.^c^

FGF-23 has also been measured in non- and pre-azotemic hyperthyroid cats. Plasma FGF-23 concentrations were higher in pre-azotemic cats than nonazotemic cats and increased following the treatment of hyperthyroidism.^d^ FGF-23 was also evaluated prospectively in nonazotemic cats. Again, it was shown that FGF-23 was higher in cats with higher creatinine values (>139.7 μmol/L [>1.58 mg/dL]). A weak relationship was found between FGF-23 and PTH. However, the positive relationships between FGF-23 and phosphate and calcitriol were not significant. Finally, as expected, there was an inverse relationship between FGF-23 and GFR (iohexol clearance).^e^ FGF-23 concentrations were also evaluated in cats before and after being fed a renal diet. FGF-23 and phosphorus concentration decreased in hyperphosphatemic cats after being fed a renal diet despite the lack of change in creatinine. In normophosphatemic cats, FGF-23 also decreased after cats were fed a renal diet despite the lack of change in phosphorus and creatinine concentration. In conclusion, feeding a renal diet is associated with a reduction in plasma FGF-23 concentration in hyper- and normophosphatemic cats with stable azotemic CKD. Therefore, dietary phosphate and protein restriction has effects on phosphate homeostasis, even when plasma phosphorus does not significantly change.^f^

### VDRA

VDRA is the term used to indicate the effects following ligand binding of a natural vitamin D compound or synthetic analogue to the VDR. Of the naturally occurring vitamin D metabolites, calcitriol has the greatest affinity for this receptor. Depending on the particular gene, VDRA can be up- or downregulating to a variety of cellular processes.

## Renoprotective Roles of VDRA in CKD Patients

Calcitriol has long been reported to provide benefits to the human uremic patient by lowering PTH concentration;^130^,^158^ this has also been reported for dogs and cats.^145^,^159^ Oral calcitriol has been shown to increase survival in human patients with CKD including those treated prior to dialysis,^150^,^151^ and in one placebo-controlled study in dogs with CKD as described earlier.^b^ Various studies that show increased survival can be attributed to the salutary effects of extrarenal tissue VDRA, intrarenal VDRA, and benefits of lowered PTH synthesis and secretion following VDRA in the PTG.

The antiproteinuria effects of vitamin D analogs are of crucial significance because proteinuria is a major risk factor for the progressive decline of renal function in dogs and cats.^160^,^161^ Podocytes are critically important in overall glomerular function and structure. Injury to podocytes commonly leads to proteinuria^62^ and glomerulosclerosis.^62^,^63^ Podocytes have VDR, which is markedly upregulatable,^162^,^163^ and the podocyte nucleus has a VDRE (vitamin D response element) in its DNA near the promoter start site for the nephrin gene.^164^ Calcitriol stimulates nephrin mRNA and protein;^164^ nephrin is an essential protein in the slit pore membrane of the glomerulus. The slit pore protein podocin was also upregulated by VDRA,^57^,^165^ and a marker of podocyte injury, desmin, was lowered by calcitriol in one model of CKD in rats.^165^ It is widely speculated that podocytes are a major renoprotective site for VDRA such as calcitriol.^64^ More studies in rats showed that calcitriol decreased podocyte hypertrophy and podocyte loss;^60^ podocyte apoptosis was also ameliorated by calcitriol.^61^

**Figure 10:**
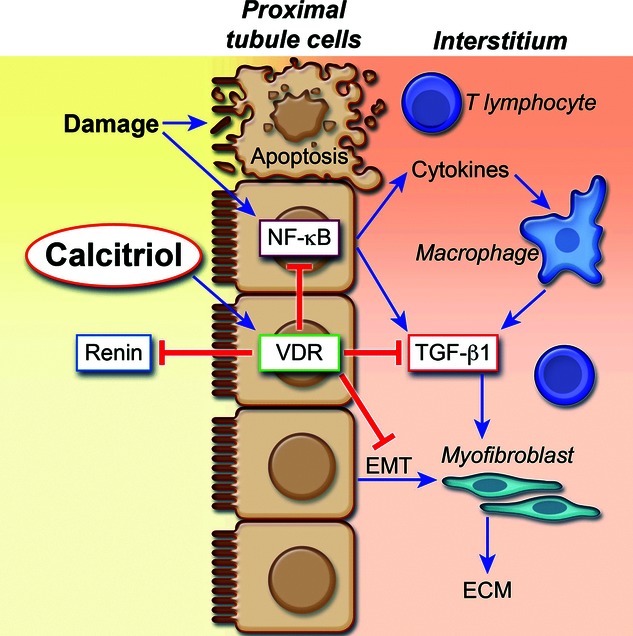
Damage to tubular cells causes both apoptosis and activation of NF-κβ, which in turn has numerous effects, mediated through inflammatory and immunomodulatory cytokines acting on mononuclear cells of lymhocytic and macrophage lineages. NF-κβ also induces formation of TGF-β the major driving cytokine of fibrogenesis acting on myofibroblasts to produce extracellular matrix (ECM). The actions of calcitriol or other VDRA on the VDR have 4 main consequences illustrated. (1) Liganded VDR blocks transcription of the Renin gene commonly by over 90% thus slowing RAAS activity, (2) Liganded VDR complexes with NF-κβ disallowing its transcription factor function including numerous cytokine regulations TGF-β being an important one decreasing fibrogenesis, (3) Liganded VDR has direct effects to repress TGF-β formation by genetic regulations, and (4) Liganded VDR acts to decrease the epithelial-to-mesenchymal transition (EMT) thus decreasing formation of myofibroblasts from epithelial cells a process active in any renal injury. (Adapted from: Liu Y. Cellular and molecular mechanisms of renal fibrosis. Nat Rev Nephrol 2011; **7**(12): 684–696.)

Fibrosis as either glomerulosclerosis or tubulointerstitial fibrosis is a common outcome of CKD.^166^ Figure 10 illustrates mechanisms likely to operate in the initiation and the progression of CKD and also the steps where calcitriol may interrupt these events. Calcitriol in physiologic doses interfered with glomerular proliferation and growth, lessening development of glomerulosclerosis in a rat model.^71^ Calcitriol and analog treatment of an experimental glomerulonephritis model in rats inhibited mesangial cell proliferation, glomerulosclerosis, and albuminuria.^167^ Exposure to activated vitamin D compounds induces an antifibrotic growth factor that blocks matrix production by interstitial fibroblasts as well as myofibroblastic activation.^68^ Calcitriol induces increases of antifibrotic factors such as matrix metalloproteinase (MMP) 8, which cleaves collagen and BMP7, a bone morphogenic protein, that is a potent antagonist of the fibrosis stimulated by transforming growth factor beta (TGF-β). Calcitriol also represses formation of TGF-β,^68^ the best known promoter of fibrosis as well as SERPINE 1 another prominent promoter of fibrosis.^69^,^67^

The renin-angiotensin-aldosterone system (RAAS) is a major mediator of progressive renal injury in CKD.^72^ The RAAS system is present entirely within the kidney. Juxtaglomerular cells are the most active in initiating RAAS, but the system is present in most renal cells including tubular epithelia. Drugs that target the RAAS, including angiotensin-converting enzyme inhibitors (ACEI) and angiotensin II (Ang II) type 1 receptor blockers (ARB), have been shown to slow the progression of glomerulosclerosis, tubulointerstitial fibrosis, and proteinuria.^168^,^169^ Intrarenal Ang II exerts multiple effects on the kidney (eg, increased glomerular capillary pressure, induction of profibrotic and proinflammatory cytokines, promotion of inflammatory-cell infiltration, stimulation of cell proliferation and hypertrophy, and upregulation of extracellular matrix synthesis) that promote progression of renal injury.

Blockade of the RAAS with ACE inhibitors^170^ and Ang II receptor blockers^171^ is commonly used to control hypertension, proteinuria and progression of CKD. Normal feedback mechanisms activated as a consequence of these medications lead to increased renin concentration. Excess renin production diminishes effectiveness of those drugs. A profibrotic effect of high renin concentration on a (pro)-renin receptor^172^ also occurs as a negative consequence of this result.^173^ Nonsuppressed conversion of angiotensinogen to angiotensin I can overwhelm the ability of the ACE inhibitor to decrease formation of Ang II, the RAAS hormone with greatest negative consequence to the uremic patient.^174^

Calcitriol is a negative endocrine regulator of the RAAS.^175^,^176^ Calcitriol suppresses renin biosynthesis and has a protective role against hyperglycemia-induced renal injury in diabetic patients.^72^ VDRA in the kidney lessens renal fibrosis via interactions with calcitriol that dampen the RAAS.^76^ The calcitriol suppressed transcription of the renin gene^44^,^177^ was independent of both PTH and calcium.^75^ Ang II functions as a major fibrogenic cytokine stimulating epithelial-to-mesenchymal transition (EMT), TGF-β and a downstream factor, connective tissue growth factor beta (CTGF-β).^76^ Through its effects to inhibit the RAAS, calcitriol decreases production of Ang II^178^ and thus lessens these fibrogenic consequences as well as other harmful renal effects.

A glomerular mesangial or interstitial inflammatory reaction with marked involvement of macrophages and lymphocytes attends all forms of renal disease.^57^ Together with control of RAAS, the ability of calcitriol and other ligands that provide VDRA to control renal inflammation are considered hallmarks of renoprotective actions mediated through the VDR and its control of the genetic apparatus.^10^,^57^,^58^ In addition to direct effects of the VDR-ligand on genes for cytokines and interleukins that control lymphocytes, a major general anti-inflammatory action is accomplished by the complexing of the VDR-ligand to a component of a group of major transcription factors (ie, NF-κβ) regulating many immune reaction and inflammation related genes.^57^,^179^,^180^ Complexing of liganded VDR to the nuclear transcription factor NF-κβ cripples its ability to stimulate inflammation related genes.^57^,^179^,^180^ Calcitriol can suppress the induction of angiotensinogen (a prerenin step) by blocking the NF-κβ pathway with consequent slowing of RAAS, which helps control the proteinuria of diabetes mellitus.^181^

**Figure 11:**
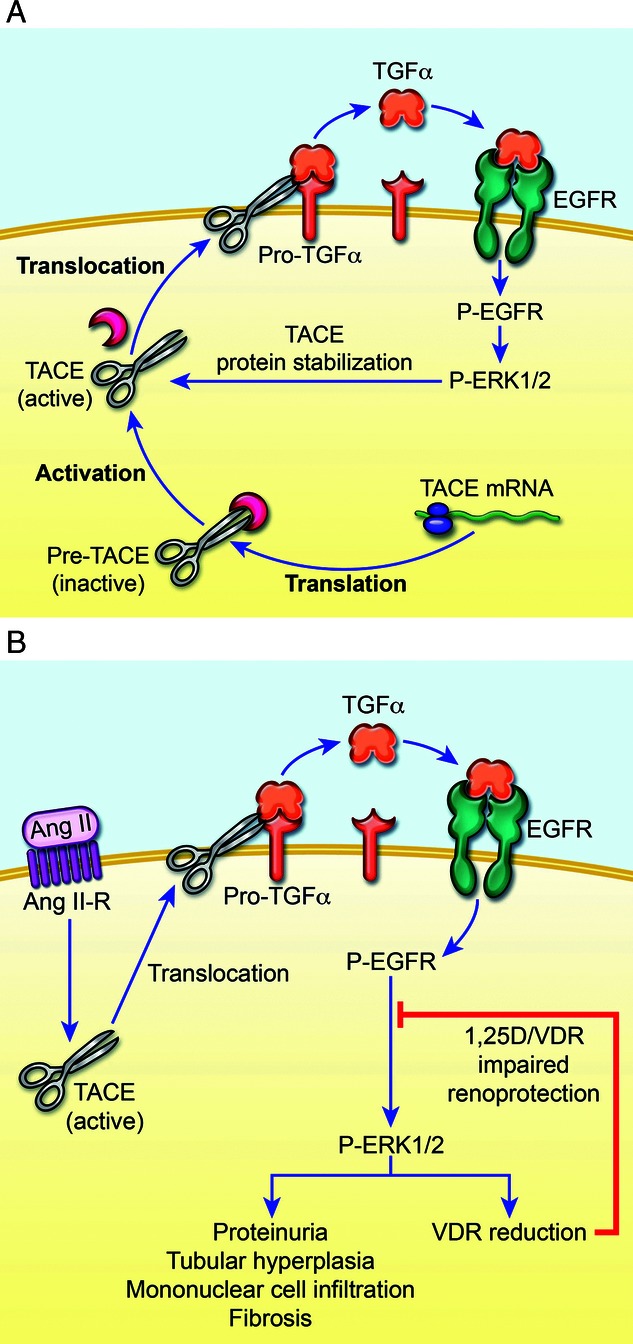
A,B. Angiotensin–II Control of TACE, TGF-α, and EGF-R in Parathyroid and Kidney (Adapted from: Dusso A, Gonzalez EA, Martin KJ. Vitamin D in chronic kidney disease. Best Pract Res Clin Endocrinol Metab 2011; 25: 647–655, and Dusso A, Arcidiacono MV, Yang J, Tokumoto M. Vitamin D inhibition of TACE and prevention of renal osteodystrophy and cardiovascular mortality. J Steroid Biochem Mol Biol 2010; 121: 193–198.) (A) Intracellular TACE (tumor necrosis factor alpha converting enzyme) translated from its mRNA in an inactive form has activation and translocation to membrane accomplished by removal of an inhibitory component. TACE in its active form is strongly stabilized by the phosphorylated EGFR (epidermal growth factor receptor) via P-ERK1/2 forming a “feed-forward” loop with TACE action via its sheddase action on Pro-TGF-α (transforming growth factor alpha [TGF-α]) generating TGF-α able to activate more EGFR and continue the cycle resulting in more generation of each participant in a “vicious cycle” with marked pathologic consequences. P-ERK ½ (extracellular signal related kinase or MAP-kinase) a general form of kinase involved in cellular effect amplifications. (B) Angiotensin II (ANG II) together with its receptor shown as the activating and translocating factor for TACE acting to generate TGF-α as the main ligand for EGFR, which when phosphorylated acts via P-ERK ½ in many pathologic roles in renal disease. P-ERK ½ diminishes the activity of calcitriol by lowering VDR levels. In parathyroids, the P-EGFR translocates to nucleus to markedly stimulate cyclin enzymes producing parathyroid hyperplasia.

The relationships of some antiproliferative (eg, PTG) and renoprotective (eg, kidney) roles of calcitriol to Ang II and epidermal growth factor receptor (EGFR)-mediated effects involved in worsening of renal failure have been of great interest.^10^,^42^,^155^ All have related to tumor necrosis factor alpha-converting enzyme (TACE), transforming growth factor alpha (TGF-α), EGFR and extracellular regulated kinase 1 and 2 (ERK 1/2) (Figure 11A and B) with the suggestion that TACE is inhibited by VDRA.^42^ The TGF-α released by TACE is a major activator of EGFR stimulating its phosphorylation (Figure 1A). Calcitriol inhibition of TACE appears to be the consequence of the impedance of the feed-forward loop between phosphorylated-ERK 1/2 (P-ERK 1/2) and TACE stabilization (Figure 11A), so that the inhibition of EGFR action by calcitriol (Figure 11B) leads to decrements of TACE. Calcitriol also inhibits the nuclear translocation of EGFR with blockade of its subsequent activation of nuclear cyclin-related enzymes of cell proliferation.^182^ Some of the “sheddase” actions of TACE liberate serum factors including tumor necrosis factor-alpha (TNF-α), intercellular adhesion molecule 1 (ICAM-1), and vascular adhesion molecule 1 (VCAM-11). Measurement of some of these serum factors has been proposed to quantitate TACE activity.^10^

Increased systemic and transglomerular blood pressure are known consequences of an activated RAAS and increased generation of Ang II, and is strongly associated with uremic progression.^183^ EGFR activation has, via the ERK 1/2 phosphorylation, multiple effects associated with uremic progression (Figure 1B) including proteinuria, glomerulosclerosis, tubular hyperplasia, mononuclear cell infiltration, and fibrosis.^155^ Linking Ang II to EGFR is critical for this theory of RAAS consequences and was documented by showing Ang II activation of TACE and facilitation of its translocation to the cell membrane (Figure 1B). TACE then releases TGF-α from its membrane protein precursor^184^ so it can activate EGFR (Figure 11A). Thus, it is not the increased blood pressure per se that causes uremic progression as had long been believed, but rather the Ang-II-associated activation of EGFR via TACE.^183^ Importantly, the P-ERK 1/2 generated causes a marked reduction in VDR (Figure 11B). Downregulation of VDR is associated with PTG hyperplasia.^39^,^185^ The pathologic sequence of events as illustrated in Figure 11A and B occur nearly identically in parathyroid cells and many kidney cells including podocytes.^40^ The CaR is decreased in hyperplastic parathyroid cells due to lack of VDR needed for its induction by calcitriol.^23^,^186^ Rescue of PTH control with calcitriol treatment during CKD is more difficult as parathyroid hyperplasia advances due to downregulation of both VDR and CaR.

Negative effects of Ang II are many, but may be attenuated by replacing lost Klotho,^81^,^187^ which Ang II downregulates.^188^ The Klotho gene and protein function as a renoprotective factor.^189^ Klotho deficits caused by effects of Ang II may be associated with vascular calcifications.^49^ Calcitriol induction of Klotho^50^,^190^ has renoprotective consequences in part by counteracting negative effects of excessive Ang II.^81^,^187^ Klotho appears to work by stabilizing integral membrane proteins, importantly ion channel proteins, in their membrane localizations.^191^ Vitamin D analogs have been used in a double-blind randomized study showing that they reduced albuminuria and inflammatory status in patients already receiving angiotensin converting enzyme inhibitor or ARB; these effects were independent of their actions on hemodynamics and PTH suppression.^192^ ARB treatment markedly increases renin concentration in the kidney.^193^ However, the combination of ARB and vitamin D analogs markedly attenuates induction of renin and Ang II.^72^

### Extrarenal benefits of VDRA

VDRA in many of the nontraditional tissues (aside from bone, kidney, intestine, and PTG) may explain beneficial effects independent from decreased synthesis and secretion of PTH. In some instances, calcitriol treatment facilitates local tissue uptake of calcidiol through as yet undefined mechanisms.^80^ At least 38 different tissues are known to contain the VDR and often these cells contain 1-α-hydroxylase enzyme that generate calcitriol locally.^82^ This is in an autocrine/paracrine manner rather than as a renal (endocrine) production of calcitriol. These cells respond to exogenous or kidney-generated calcitriol as well as to the internally generated secosteroid. These autocrine/paracrine related VDR rely critically on an adequate source of calcidiol so that calcitriol can be made internally. This helps to explain the essentiality of adequate vitamin D nutrition in patients with constrained levels of circulating calcitriol. Even in the presence of advanced renal failure, peripheral tissue generation of calcitriol can be maintained if an adequate nutritional supply of vitamin D and thus calcidiol is available.^194^,^195^ The effects of VDRA in peripheral tissues are beyond the scope of this review, though noted to be very important.

### Vitamin D and calcidiol–Emerging concepts in CKD

Survival in people with CKD has been correlated more with circulating calcidiol than with calcitriol. This observation could be attributed to a greater VDRA activation in peripheral tissues or a greater calcitriol generation when the circulating calcidiol concentration is higher. Very little calcidiol makes its way to the kidney 1-α-hydroxylase enzyme via the bloodstream by diffusion through the basolateral tubular cell membrane. The renal 1-α-hydroxylase residing in mitochondria of proximal convoluted tubules processes calcidiol bound to VDBP^10^ following glomerular filtration and tubular receptor-mediated endocytosis. The tubular scavenging receptor known as megalin^10^,^78^ works in tandem with the associated protein cubilin^77^,^78^ that delivers the calcidiol/VDBP. Megalin synthesis by renal cells is dependent upon its genetic induction by calcitriol.^10^

An 80% incidence of vitamin D deficiency in people with CKD has been reported based on measurement of calcidiol.^196^ Low concentrations of calcidiol in CKD may be the result of reduced megalin expression, which reduces calcidiol uptake into tubular cells and decreases its return to circulation (due to enhanced loss of the calcidiol–VDBP complex into urine). Progressive CKD also leads to an increase in FGF-23, which leads to enhanced activity of 24-hydroxylase, thereby increasing the degradation of calcidiol to 24,25(OH)_2_-vitamin D. Malnutrition can contribute to decreased intake of vitamin D, resulting in a lower serum concentration of calcidiol. Treatment with activated vitamin D compounds upregulates 24-hydroxylase activity, which decreases circulating concentrations of both calcidiol and the activated vitamin D compound due to increased degradation.^10^ Details from dogs with naturally occurring CKD and low calcidiol status were described in an earlier section. The degree of low calcidiol status in dogs and cats with CKD may be different in magnitude from that observed in people since commercial diets for dogs and cats contain abundant vitamin D content.^146^,^157^

Supplementation with parent vitamin D to human patients with CKD leads to improved serum calcidiol concentrations and markers of mineral and bone metabolism as well as reduces the dose of calcitriol needed.^197^ In people, calcitriol concentrations can be normalized with vitamin D supplementation when serum calcidiol concentration approaches concentrations of 100 ng/mL.^10^ In experimental dogs with mild and moderate CKD, calcidiol concentration correlated with serum calcitriol concentration. Serum calcitriol concentration could be normalized following vitamin D supplementation achieved by a 2-fold increase in calcitriol production when more calcidiol substrate was provided.^134^ Standard doses used for vitamin D replacement (to achieve a targeted calcidiol level) often do not achieve targeted concentration in people with CKD due to reduced megalin expression in the proximal tubules and the above mentioned loss of calcidiol to the urine.^10^

### Treatment protocols for patients with CKD using vitamin D compounds

**Figure 12:**
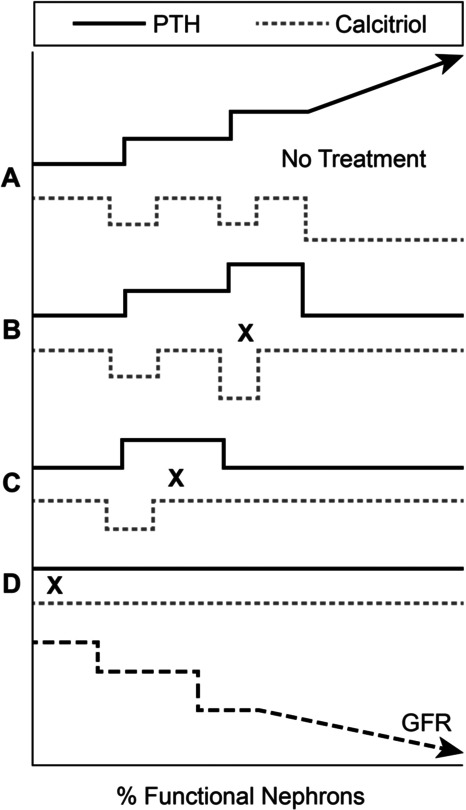
Different stages of CKD in which calcitriol treatment can be considered A = No calcitriol supplementation. Calcitriol normalizes only at the expense of elevated PTH. B = Calcitriol treatment is started at time “*x*” late enough in the renal disease when calcitriol is decreased and PTH is elevated with restoration of both to normal. C = Calcitriol treatment is started at an early enough stage where calcitriol concentrations are still normal as a consequence of the increased PTH. Calcitriol supplementation remains beneficial to maintain normal calcitriol concentrations while decreasing PTH. D = Calcitriol treatment is started very early in the course of progressive nephron loss, prior to either PTH increase or calcitriol decrease as a preventative step.

**Figure 13:**
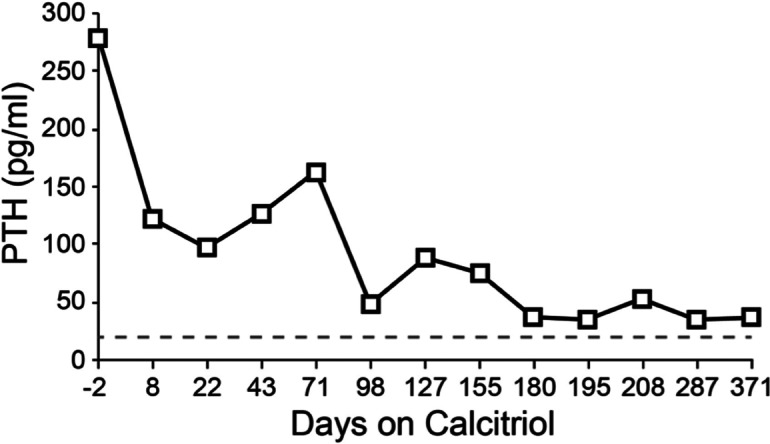
Time sequence for PTH suppression in a cat with CKD. A standard dose of 2.5 ng/kg once daily was given orally.

Treatment with activated vitamin D compounds provides renoprotective effects and extrarenal effects that are known to be beneficial in people. Most of our treatment recommendations in veterinary medicine have been extrapolated from findings in experimental animals or from human medicine since controlled outcome studies of VDRA in dogs and cats as the target species are lacking. VDRA with calcitriol has traditionally been advocated for its ability to decrease PTH synthesis and secretion. VDRA in the kidney exerts salutary effects to lessen inflammation and fibrosis through a wide variety of mechanisms including dampening of the local renal RAAS and reduction of the generation of crucial cytokines.^198^ VDRA in peripheral tissues also provides benefits to those tissues. Figure 12 illustrates different time-points during CKD in which calcitriol treatment can be considered and the expected responses. Figure 13 shows the time sequence for PTH suppression in a cat with CKD treated with oral calcitriol. PTH in healthy cats is close to 20 pg/mL using this assay. Notice the gradual decline in circulating PTH over several months. It took about 4 months of treatment to return PTH to a targeted concentration. It is our impression that cats can take longer than dogs to achieve adequate control of PTH following the calcitriol treatment.

An overarching principle of initial treatment is to reduce the burden of phosphorus retention within the body. The serum phosphorus level has been shown to be predictive of survival in cats with CKD. For each 1 mg/dL increase in serum circulating phosphorus, there was a 12% increase in the risk in death.^199^ Less phosphorus retention relieves the inhibitory effect of phosphorus on renal 1-α-hydroxylase activity, resulting in the increased production of endogenous calcitriol and subsequent inhibition of PTH synthesis. Pi-retention promotes PTH synthesis by stabilization of mRNA in the PTG cell^200^ and by stimulating parathyroid hyperplasia.^39^ PTG hyperplasia is mediated in part by increased TGF-α and EGFR interactions that occurs secondary to progressive phosphorus retention. The first step in treatment is to relieve phosphorus retention by dietary restriction of phosphorus (Table 2). Dietary phosphorus restriction alone may be capable of decreasing PTH or preventing its increase in early stages of CKD in the cat and dog. Diet alone will become ineffective to control PTH as CKD advances especially as serum phosphorus concentration increases. Correction of hyperphosphatemia normalized PTH in many early uremic cats,^144^ but was successful in only a portion of uremic dogs, many of which required calcitriol to normalize PTH.^159^ Step 2 is to administer intestinal phosphate binders in food (salts of aluminum, lanthanum or sevelamer, or calcium in hypocalcemic patients) to achieve a specific targeted serum phosphorus or PTH. Specifics as to the use of intestinal phosphate binders are beyond the scope of this review, but a report that sevelamer in contrast to lanthanum interfered with absorption of orally administered calcitriol^201^ makes treatment with sevelamer less desirable in patients treated with calcitriol. Calcium-based intestinal phosphate binders are now rarely used in human nephrology in part because of the increased use of VDRA with calcitriol and concerns for development of vascular calcifications.^202^

**Table 2:** Recommendations for calcitriol therapy

(1) Obtain serum phosphorus control (ideal level: 1.5–1.8 mmol/L [4.5–5.5 mg/dL])
(a) Low phosphorus diet
(b) Use phosphorus binders (give with food divided into 2 to 3 times daily)
(i) Aluminum hydroxide–use up to 100 mg/kg/day (consider measuring plasma aluminum level when using higher doses)
(ii) Lanthanum carbonate–12.5–25 mg/kg/day
(iii) Sevelamer hydrochloride–100–160 mg/kg/day
(iv) Avoid calcium-containing phosphorus binders
(2) After 2 weeks or more of phosphorus control, submit serum PTH and assess secondary renal hyperparathyroidism. Calcitriol is indicated whether or not PTH is elevated (renoprotective mechanisms independent of PTH)
(3) Calcitriol administration (normophosphatemic and normocalcemic [ionized calcium])
(a) Start at 2.5 to 3.5 ng/kg/day apart from meals and check ionized calcium 10 days later
(b) Recheck PTH level in 4 weeks
(c) Increase calcitriol dose by 1 ng/kg/day until PTH is controlled (within normal range or ionized hypercalcemia occurs.
(d) Recheck ionized calcium 10 days after increasing the dose and once monthly.
(e) Recheck PTH every 4 weeks until PTH control is achieved, then every 3 months or when azotemia worsens.
(4) Calcitriol administration (intermittent twice weekly dose)
(a) Use 3.5 times daily dose (typically 9 ng/kg given orally every 3.5 days.
(b) Dose on empty stomach 4 h before and 2 h afterward to avoid transcaltachia induced transient hypercalcemia.

Optimal PTH control in dogs with CKD is best achieved when a target serum phosphorus of less than 1.5 mmol/L [4.5 mg/dL]^110^ or a concentration of serum phosphorus in the middle of the reference interval is achieved. Return of serum phosphorus to within the normal reference interval is an initial goal, but does not guarantee the adequate control of PTH production. Very high serum phosphorus concentration can lower iCa through mass-law effects providing a stimulus for increased PTH secretion. The combination of diet and intestinal phosphate binders can control renal secondary HPTH early on, but escape can be expected as CKD progresses.

### Calcitriol treatment strategies

Since VDR expression is downregulated in CKD, higher concentrations of circulating calcitriol are required to maintain VDR expression in these tissues.^33^ Exogenous calcitriol treatment corrects the absolute or relative calcitriol deficits that exist in advancing CKD. Traditionally, calcitriol has been administered to dogs and cats with CKD to restore PTH to lower concentrations. Calcitriol can also be given to prevent the development of renal secondary HPTH and PTG hyperplasia for CKD patients with PTH within the reference interval. It can be difficult to exclude renal secondary HPTH even in those with normal reference interval for PTH concentration, since this range may be large and PTH may already be increasing, but it would not be possible to know this without previous PTH concentration for comparison. Calcitriol is uncommonly specifically prescribed for the patient that has clinical signs secondary to persistent ionized hypocalcemia to increase iCa. VDRA by calcitriol in peripheral tissues provides salutary effects that are increasingly recognized in people with CKD.

Calcitriol is not available as a commercial product in a formulation suitable for administration to most dogs and cats. The human formulation (Rocaltrol)^g^ provides either 0.25 or 0.50 μg (250 or 500 ng) in each capsule; pediatric oral solution provides 1 μg/mL (1000 ng/mL). Calcitriol can be prepared by compounding pharmacies experienced in this type of dilution in pharmaceutical oil with appropriate preservatives; reformulation errors occasionally occur, which can render the preparation less effective to useless, or toxic (hypercalcemia). It is sometimes more cost effective to directly dose the oral solution form of calcitriol (no reformulation) though the small doses needed for smaller patients are difficult to accurately measure in dose syringes. For example, a 20-kg dog that is to receive 2.5 ng/kg daily dosing would need 50 ng daily or 0.05 mL of the oral solution. The same dog given 9 ng/kg twice weekly (see intermittent dosing below) would need 180 ng twice weekly or 0.18 mL twice weekly. For cats, direct dosing is impossible and the use of a competent compounding pharmacy is mandatory. Alphacalcidol (1-α-(OH)-vitamin D_3_) rather than calcitriol is available to prescribe as an active vitamin D metabolite in some parts of the world. Alphacalcidol is converted rapidly in the liver to 1,25(OH)_2_-vitamin D_3_, the active metabolite calcitriol. Capsules (One-Alpha)^h^ containing either 1.0 μg (1000 ng) or 0.25 μg (250 ng) of alphacalcidol (1-α-hydroxyvitamin D_3_) are available. Slightly higher doses compared to calcitriol may be needed to compensate for some loss during the 25-hydroxylation process.^203^–^205^

Serum phosphorus should be less than 1.9 mmol/L [<6.0 mg/dL] before starting calcitriol treatment and thereafter to ensure calcitriol efficacy in PTH suppression. Calcitriol enhances the absorption of both calcium and phosphorus across the intestine so it is important to not increase the risk for soft tissue mineralization. The calcium × phosphorus product should remain less than 55 mg^2^/dL^2^ according to KDOQI guidelines for human nephrology, but there is little evidence to support this concept.^206^ This target is often achieved by dietary phosphorus restriction and the use of intestinal phosphate binders. If hyperphosphatemia develops during calcitriol treatment, due to further loss of renal excretory function or less likely more intestinal absorption, it is necessary to increase the extent of dietary phosphate restriction and/or to increase the dose or class of intestinal phosphate binder with use of a combination of binders (eg, aluminum, lanthanum, and sevelamer carbonate) thus minimizing any toxic effects of each individual class of binder. In some patients, it appears that the development of hyperphosphatemia has rendered previously therapeutic doses of calcitriol ineffective at suppressing PTH (serum phosphorus > 2.6 mmol/L [> 8.0 mg/dL]). This is likely due to a combination of decrements in iCa from mass-law interactions, in that calcium is needed synergistically for calcitriol to exert genomic effects within the parathyroid cell, and to destabilize PTG mRNA that leads to decreased PTH synthesis. Although calcium destabilizes PTH mRNA, phosphorus stabilizes PTH mRNA, which favors PTH synthesis.^200^,^207^ In addition to beneficial effects on PTH control, it is advisable to return serum phosphorus to normal concentration in an effort to avoid soft tissue mineralization (including the kidney).^159^

Hypercalcemia is a very uncommon side effect when low-dose oral calcitriol therapy is used. Hypercalcemia is more commonly associated with the use of calcium-containing intestinal phosphate binders, especially calcium carbonate while receiving calcitriol.^208^ Sometimes hypercalcemia develops when calcitriol has been overdosed, or if the patient is particularly sensitive to standard low doses of calcitriol. The occurrence of hypercalcemia can be minimized by giving calcitriol at night-time on an empty stomach, which primes the enterocytes for calcium absorption at a time of minimal calcium exposure within the intestinal lumen. Use of intermittent dosing of calcitriol has been useful in the authors’ experience in amelioration of hypercalcemia resulting from daily administration of calcitriol at higher doses. If hypercalcemia is caused by the administration of excessive calcitriol, the serum total and iCa concentration declines during the week after the discontinuation of calcitriol due to calcitriol's short half-life in blood (4–6 hours) and a biological half-life of 2–4 days.^4^ A lower dose of calcitriol or use of intermittent dosing is subsequently prescribed if the serum calcium declines after discontinuation of calcitriol. An estimated 10%–15% of dogs develop mild-to-moderate serum total hypercalcemia as a consequence of their chronic kidney failure (on no calcitriol treatment) for a variety of possible mechanisms; in these instances, iCa is often normal or low.^209^ Total calcium is even more unpredictable in cats.^210^ Therefore, it is imperative to measure iCa when increases in serum total calcium are observed in order to determine if this type of calcemia is dangerous or not to the patient. Serum total calcium concentration will not decline when calcitriol is discontinued if the increased serum total calcium concentration is due to the increased complexed calcium fraction as is frequently the case in dogs with CKD.^211^ Malignancy-associated hypercalcemia should also be considered if iCa persists after discontinuing calcitriol and calcium-containing medications. Aluminum-induced hypercalcemia is a consideration for patients receiving long-term aluminum salts for intestinal phosphate binding, but is rare. This has been reported after parenteral administration of aluminum.^212^ However, iCa was not measured in this study and a different study actually showed that iCa decreased slightly after parenteral infusion of aluminum in dogs while the total calcium increased.^213^ Hypophosphatemia and low PTH have been rarely described in cats and dogs on a low phosphate diet that developed ionized hypercalcemia; the hypercalcemia resolved when fed a higher dietary phosphorus.^111^,^136^ The mechanism for the hypercalcemia in this instance may have been due to adynamic bone due to excessive suppression of PTH. Other hypotheses include low serum phosphorus concentration leading to stimulation of calcitriol production and changes in calcium/phosphorus balance.^136^ This is neither common nor important in the typical dog or cat with CKD; however, it may explain the lack of success of renal diets in lowering iCa in feline idiopathic hypercalcemia.

The tradtional goal of low-dose calcitriol treatment during CKD is to provide adequate suppression of PTH without inducing ionized hypercalcemia. VDRA following exogenous calcitriol supplementation likely provides benefits to peripheral tissues in veterinary patients as has been shown in people with CKD. Periodic re-evaluation of serum PTH concentrations should be performed at 1, 3, and 6 months of calcitriol treatment. PTH is best interpreted with serum iCa concentration measured at the same time. This is to ensure that the prescribed dose of calcitriol is effective in maintaining near-normal PTH concentrations, but that ionized hypercalcemia does not develop, and that oversuppression of PTH does not occur. When serum phosphorus continues to be rigorously maintained at 1.9 mmol/L [6.0 mg/dL] or less, the probability for future PTH escape is low. Further reduction of serum phosphorous to below 1.5 mmol/L [<4.5 mg/dL] may also be helpful in rendering the administered calcitriol more effective in suppression of PTH. This PTH control can be considered a form of proxy for the host of calcitriol's renoprotective benefits. There are several factors associated with inadequate control of renal secondary HPTH during calcitriol therapy (Table 3).

**Table 3:** Factors hindering adequate control of secondary hyperparathyroidism during calcitriol treatments

↓ Ionized calcium
Calcium with its associated transcription factor must bind to its DNA binding site in the PTG cell nucleus in order to fully allow the silencing effect of calcitriol to decrease transcription of the PTH mRNA and thus the synthesis of PTH.
↑ Phosphorus.
↑ Stabilization of mRNA in PTG nucleus favors PTH synthesis.
↓ Ionized calcium in those with very high phosphorus.
↑ TGF-α & EGFR to stimulate PTG hyperplasia
Severe parathyroid gland hyperplasia
↓ VDR expression and decreased CaR expression→ Altered “set point” dynamics
Uncontrolled metabolic acidosis
↓ Synthesis of endogenous calcitriol from 25(OH)-vitamin D
↓ Circulating 25(OH)-vitamin D – from ↑ loss into urine (↓ megalin)
Less substrate for synthesis of calcitriol

## Daily Dosing

Supplementation with calcitriol in CKD was initially designed as a daily therapy for life in veterinary patients as long as serum phosphorus remains within reference interval and serum iCa concentration does not increase above the reference interval. The majority of naturally occurring early CKD and creatinine concentrations within the range of 176.8 – 221 μmol/L [2–2.5 mg/dL] will have HPTH effectively reversed or prevented by doses of calcitriol between 2.5 and 3.5 ng/kg/day. Doses lower than 2.5 ng/kg are rarely used, and occasionally a dose as high as 6 ng/kg/day is used when lower doses do not succeed in lowering PTH. After receiving the initial dose for 2 months, a recheck of serum PTH concentration will indicate if an incremental calcitriol dosage increase is necessary.

### Intermittent dosing

Intermittent dosing of calcitriol is a method of calcitriol treatment designed to deal with patients that develop hypercalcemia while on daily calcitriol treatments. This method of dosing is less stressful for many cats compared to daily dosing. Twice weekly calcitriol at 3.5 times the daily dose is given instead of the daily average dose of 2.5 ng/kg, so that the same total nanogram per week is administered. This allows a smaller percentage of the intestinal epithelial cells to become programmed for calcium absorption while still being able to decrease synthesis of PTH.^214^ For example, a 10-kg dog that received daily calcitriol at 2.5 ng/kg (25 ng/day) would get 87.5 ng on the night of day 1 (ie, Wed) and also on the morning of day 4 (ie, Sun), using the intermittent dosing method. Intermittent dosing protocols could be used instead of daily calcitriol as the initial therapy if owners can reliably maintain the dosing interval of every 3.5 days. PTG cells dosed twice weekly have been shown to effectively control circulating PTH concentrations for 4 days.^215^ Other slowly replicating target cells are likely to respond the same so that dosing calcitriol at 3.5 times the daily dose every 3.5 days provides effective drug action at those cells.

In people, it has been shown that an intermittent calcitriol dosing scheme is more effective in decreasing chances of hypercalcemia than dosing daily.^216^ However, this may not be true for patients undergoing peritoneal dialysis, especially those that are receiving high doses of calcium salts.^217^

### Pulse dosing

The pulse-dosing method for calcitriol treatment can be utilized in advanced HPTH (PTH concentration 10 times normal or greater) as an initial treatment, or is more likely used after a patient fails to decrease PTH adequately following a daily or intermittent calcitriol dosing protocol. The rationale for pulse dosing is to allow much higher calcitriol doses and peak blood calcitriol concentration in an attempt to upregulate the number of calcitriol receptors in the parathyroid cells, without generating hypercalcemia that would be seen with daily dosing at a high level. With pulse dosing, calcitriol is given twice weekly at night on an empty stomach (ie, gastrointestinal tract containing minimal calcium) to minimize calcium absorption. A starting pulse dose is 20 ng/kg orally twice weekly. Serum calcium is measured one and 2 days after the third dose to assess potential development of hypercalcemia. It is preferable to measure serum iCa. Serum total calcium (tCa) can be measured and used in comparison to serum tCa measured prior to treatment. It is conceivable that some hypercalcemia can occur rapidly as a result of transcaltachyia, a direct cell membrane effect of calcitriol on the intestinal epithelium independent of any genomic effects that calcitriol exerts later. This is transitory and of little significance. PTH is remeasured after 1 month of calcitriol, and if not significantly suppressed, the dosage of calcitriol may be incrementally (5 ng/kg) increased, again checking for hypercalcemia as above after any increase in the calcitriol dose. Once PTH is decreased to near the normal range and is maintained for at least 2–3 months, it is assumed that the hyperplastic PTGs have regressed, and pulse dosing can be replaced by daily doses of calcitriol typically in the usual dose range of 2.5–3.5 ng/kg or its equivalent twice weekly.

## Calcitriol Analogues

So-called “noncalcemic” calcitriol analogues have been advocated to be superior to native calcitriol. The development of these compounds has been undertaken in an effort to find compounds that will suppress PTH in CKD patients with less risk for development of hypercalcemia than that with calcitriol. This was most important when calcium-containing gut phosphorus binders were widely used. With the advent of lanthanum carbonate or sevelamer carbonate, which is now widely used, this is less of an issue. These vitamin D analogues are catabolized much more quickly than calcitriol and have a very short half-life in blood due to diminished binding to vitamin D binding protein. The short half-life lessens the chance for hypercalcemia to develop from intestinal epithelial cell programming as only newly formed villar cells are affected by vitamin D metabolites.^214^ Various alternative mechanisms have been considered for “noncalcemic” actions of some calcitriol analogues, but the evidence for these is very weak.^218^ One of the potential down-sides to the use of these analogues is that because they bind somewhat poorly to the calcitriol receptor (VDR), large doses must be given to achieve the required level of PTH suppression; these large doses induce not only their own destruction, but also the catabolism of native calcitriol. Calcitriol analogues are much more expensive to use than calcitriol, which presently limits their application in veterinary medicine.

The vitamin D analogues used in clinical human nephrology include paricalcitol (19-nor-1a,25(OH)_2_-vitamin D_2_; Zemplar),^i^ 22-oxacalcitriol (maxacalcitol: 22-oxa-1a,25(OH)_2_-vitamin D_3_),^j^ and doxercalciferol (1a-(OH)-vitamin D_2_; Hectorol).^k^ Paricalcitol^i^ and 22-oxacalcitriol^j^ require no further metabolism to be active, which allows them to bind directly to the VDR. Doxercalciferol^k^ is a pro-drug that requires liver metabolism to add the 25(OH) group to provide the active metabolite 1,25(OH)_2_-vitamin D_2_; alfacalcidol^h^ requires this same liver 25-hydroxylation.^219^

The effects of IV and oral 1,25-dihydroxy-22-oxavitamin D (OCT) have been studied in dogs with experimental CKD and found to potentially have a wider therapeutic window than that of calcitriol (at very high dosages). Serum PTH was suppressed by 67% at a daily oral dose of 0.05 μg/kg (50 ng/kg); 0.025 μg/kg (25 ng/kg) maintained PTH within the normal range without hypercalcemia for a 4-week period.^220^ The cost to use this approach is very high.

Doxercalciferol^k^ is reputedly less calcemic than calcitriol, probably explained by less binding to VDBP in blood that accelerates its catabolism. This short half-life is responsible for less calcemia as discussed previously. 22-Oxacalcitriol has a much shorter half-life than calcitriol with one of the shortest half-lives of all the vitamin D analogues at less than 30 minutes. Paricalcitol^i^ is another widely studied and prescribed vitamin D analogue used in human medicine. For this compound, the noncalcemic effects are less well understood as its half-life is comparable to calcitriol while suppressing PTH as well as or slightly better than calcitriol in one study in hemodialysis patients albeit at considerably higher doses.^221^

Properly used, calcitriol as the natural hormone is still the gold standard for replacement therapy with activated vitamin D metabolites in the patient with CKD in which PTH must be suppressed. Calcitriol is also considerably less expensive that calcitriol analogues. Based on the emerging information about low calcidiol status in humans with CKD and a preliminary report in dogs with CKD (discussed earlier), it is possible that veterinary patients with CKD would also benefit from vitamin D treatment (parent hormone). Combination therapy for CKD patients with parental and fully activated vitamin D is common in human nephrology and deserves further study in veterinary patients with CKD.

### Monitoring therapy in the CKD patient

Surveillance for routine veterinary CKD patients involves the measurement and analysis of serial serum BUN, and creatinine, phophorus, and tCa. This surveillance is to provide information about the rate of progressive loss of excretory renal functions. Attention to calcium and phosphrous status is even more important during treatment with vitamin D and activated vitamin D compounds in order to provide early detection for the development of hypercalcemia or hyperphosphatemia.

Calcium fractions vary widely in disease and cannot be predicted accurately on the basis of tCa measurement.^144^,^149^,^209^–^211^,^222^–^226^ For accurate assessment of calcium status, iCa must be measured directly. iCa measurement has been shown to be superior to serum tCa measurements in many conditions, especially in HPTH, kidney disease, hypoproteinemia and hyperproteinemia, acid-base disturbances, and critical illnesses.^144^,^149^,^209^–^211^,^222^–^226^ Changes in the magnitude of serum protein concentration, individual protein binding capacity and affinity, serum pH, and complexed calcium all interact to determine the iCa concentration, independent of the tCa concentration.^209^,^210^,^222^

Most dogs and cats with azotemic CKD have normal serum tCa concentrations. The finding of hypercalcemia and primary renal azotemia poses a special diagnostic problem because hypercalcemia, if ionized, can cause renal failure or develop as a consequence of CKD. The incidence of increased tCa increases with severity of azotemia. Serum PTH concentration is often increased in patients with hypercalcemia related to kidney failure, and these animals must be differentiated from those with primary HPTH.^110^,^144^ Serum iCa concentration is increased in primary HPTH but is usually normal or low in patients with CKD.^110^,^209^,^227^ Deleterious effects of hypercalcemia occur in patients with renal failure only if it is associated with increases in serum iCa concentration. The mechanisms of increased serum tCa concentration in CKD have not been well characterized. In dogs with azotemic CKD and increased serum tCa, the iCa concentration is frequently normal, attributed to an increase in the complexed calcium fraction.^211^ In CKD, organic anions such as citrate, phosphate, lactate, bicarbonate, and oxalate are capable of complexing with calcium. Fasting serum samples collected at the same time in the morning are advised.

Clinical laboratories typically measure inorganic phosphate (Pi) in serum. Normal serum Pi is in the 0.9–1.8 mmol/L [3.0–5.5 mg/dL] range in dogs and cats that have finished bone growth. Normal reference intervals from commercial laboratories often include values from young growing animals and list higher upper limits, up to 2.6 mmol/L [8.0 mg/dL] in dogs, so it is important to factor this in when deciding whether hyperphosphatemia is present or not.^228^ Hyperphosphatemia of the young is attributed in part to increased concentration of growth hormone. Blood samples should be collected from fasting animals since a carbohydrate meal or infusion of dextrose can decrease the serum Pi and protein intake can increase serum Pi.^4^

PTH should also be measured in patients receiving calcitriol to assess the level of control of PTH production. Ideally, PTH should be lowered to slightly above or within the reference interval. Though intact PTH measurement was validated and reported for clinical use in dogs^229^ and cats,^230^ these assays are no longer commercially available. Whole PTH assays have been validated for dogs 231 and cats 232 and a whole PTH assay is currently commercially available for PTH measurement in veterinary patients.^l^ It is important to measure both serum iCa and PTH on the same sample to determine whether an increase or decrease in dose of calcitriol is needed. In addition, it may be beneficial to measure circulating calcitriol and calcidiol in these patients. Ideally, baseline concentration prior to treatment would be available for comparison. Measurement of calcidiol is readily available for veterinary patients. As serum calcitriol measurement becomes more routinely available from veterinary diagnostic laboratories, measurement at baseline and during treatment will be of some value to accurately assess calcitriol status. In the future, FGF-23 measurement may also become important in early detection of CKD, and beneficial in the monitoring of therapy.

## Conclusions

Renal secondary HPTH is common in both dogs and cats with CKD. Though secondary renal HPTH helps to maintain circulating phosphorus within tolerable concentration during early stages of CKD, higher concentrations of PTH eventually become maladaptive to a variety of organs including the kidneys. The frequency of increased PTH in CKD increases with the severity of CKD based on IRIS staging, but has been observed early in some dogs with IRIS stage 1 CKD. Increased PTH in both dogs and cats can occur in the face of normal serum phosphorus; serum phosphorus is an insensitive marker for phosphorus metabolism. Absolute or relative deficits of calcitriol are central to the genesis of renal secondary HPTH. Phosphatonins are important in the regulation of circulating phosphorus in health and during CKD. The most important phosphatonin is FGF-23 that lowers circulating phosphorus through effects on the kidney that cause phosphaturia and the following decreased renal calcitriol synthesis through its actions on the renal hydroxylase system. FGF-23 concentrations have been associated with some studies of survival in human CKD patients; these kinds of studies have not yet been reported in dogs or cats. Circulating FGF-23 can be decreased in CKD patients following treatment using dietary phosphorus restriction and intestinal phosphorus binding agents. Tight control of serum phosphorus remains central in the treatment of dogs and cats with CKD for its known effects associated with decreased circulating PTH and that extend patient survival independent of PTH. Also, greater relief of total body phosphorus burden allows more renal synthesis of calcitriol. Recent studies in dogs document that those with serum phosphorus within the upper third of the normal reference interval often have increased PTH; CKD dogs with serum phosphorus less than 1.5 mmol/L (<4.5 mg/dL) often have PTH concentrations within the reference interval.

Treatment of CKD with activated vitamin D metabolites has been considered important for decades in human nephrology. Initially it was thought that most of the benefits derived following treatment with activated vitamin D metabolites in CKD was from its PTH lowering effects. Though control of PTH in CKD is important, it is becoming increasingly apparent that renoprotective effects independent of PTH or calcium are quite important. Calcitriol's renoprotective actions following intrarenal VDRA are associated with important downregulation of RAAS activity. Very recently, the role for calcitriol to suppress TACE activation during CKD has been advocated. Calcitriol treatment of dogs with CKD extends the lifespan and stabilizes excretory renal function in dogs, similar to effects seen in predialysis human patients with CKD. Long-enough trials to determine any benefits for CKD cats treated with calcitriol have yet to be conducted. The importance for treatment with parent vitamin D has become increasingly understood both in its role to provide adequate substrate for activation of calcitriol and for its local tissue benefits as calcidiol enters cells. The supportive role that calcitriol exerts for megalin-induced luminal renal tubular internalization of calcidiol/VDBP and albumin is becoming more well known; calcitriol deficits can lead to low concentrations of circulating calcidiol due to its loss in urine and may contribute to albuminuria. Vitamin D status of CKD dogs based on calcidiol concentrations is often poor as is similarly true in humans with CKD. Studies to determine the effect of parent vitamin D supplementation to dogs or cats with CKD have yet to be undertaken. It is common for humans with CKD to be treated with both parent vitamin D and activated vitamin D compounds. Further evidence-based medicine outcomes using survival and preservation of excretory renal function endpoints following treatment with various parent vitamin D and active vitamin D metabolites are needed. Such studies should be performed alone and in combination with other standard treatment using RAAS inactivation and targeted serum phosphorus control.

## Footnotes

a How KL, Hazewinkel HA, Mol JA. Photosynthesis of vitamin D in the skin of dogs cats and rats. Vet Q. 1995; 17 Suppl 1: S29.

b Polzin D, Ross S, Osborne C, Lulich J, Swanson L. Clinical benefit of calcitriol in canine chronic kidney disease. J Vet Intern Med. 2005; 19: 433.

c Geddes RF, Finch NC, Elliott J, Syme HM. Fibroblast growth factor 23 (FGF-23) in feline chronic kidney disease. J Vet Intern Med. 2011; 25: 720–1.

d Williams TL, Elliott J, Syme HM. Changes in plasma fibroblast growth factor-23 (FGF-23) concentrations after treatment of hyperthyroidism (HTH) in cats with variable renal function. J Vet Intern Med. 2011; 25: 678.

e Finch NC, Geddes RF, Syme HM, Elliott J. FGF-23 – mediator of renal secondary hyperparathyroidism or a marker of glomerular filtration rate (GFR) in cats? J Vet Intern Med. 2011; 25: 720.

f Geddes RF, Elliott J, Syme HM. Feeding a renal diet is associated with a reduction in plasma fibroblast growth factor 23 (FGF-23) in cats with stable chronic kidney disease (CKD). J Vet Intern Med. 2012; 26: 798.

g Rocaltrol, calcitriol, Roche Laboratories Inc., Nutley, NJ.

h One-Alpha, alfacalcidol, LEO Pharmaceutical Products, Ballerup, Denmark.

i Zemplar, paricalcitol, Abbott Laboratories, North Chicago, IL.

j Oxarol, maxacalcitol, 22-oxacalcitriol, Chugai Pharmaceutical Co., Ltd., Tokyo, Japan.

k Hectorol, doxercalciferol, Genzyme Corporation, Cambridge, MA.

l Diagnostic Center for Population and Animal Health, Endocrine Diagnostic Section, Michigan State University, Lansing, MI.

## Abbreviations

ARB: angiotensin II type 1 receptor blockers
Ang II: angiotensin II
BMP: bone morphogenic protein
CaR: calcium sensing receptor
CKD: chronic kidney disease
CTGF-β: connective tissue growth factor-beta
DHT: dihydrotachysterol
ECF: extracellular fluid
EGFR: epidermal growth factor receptor
EMT: epithelial-to-mesenchymal transition
ERK 1/2: extracellular regulated kinase 1 and 2
FGF-R: fibroblast growth receptor
FGF-23: fibroblast growth factor-23
GFR: glomerular filtration rate
HPTH: hyperparathyroidism
IRIS: International Renal Interest Society
iCa: ionized calcium
MEPE: matrix extracellular phosphoglycoprotein
MMP: matrix metalloproteinase
NF-κβ: nuclear factor kappa-beta
Pi: inorganic phosphate
PTG: parathyroid gland
PTH: parathyroid hormone
RAAS: renin-angiotensin-aldosterone-system
tCa: serum total calcium
TGF-β: transforming growth factor-beta
TACE: tumor necrosis factor alpha converting enzyme
TGF-α: transforming growth factor-alpha
TRPV5: transient receptor potential vanillinoid 5
UV: ultraviolet
VDBP: vitamin D binding protein
VDR: vitamin D receptor
VDRA: vitamin D receptor activation
VDRE: vitamin D response element
1,25(OH)_2_-vitamin D: calcitriol
25(OH)-vitamin D: calcidiol
XLH: X-linked hypophosphatemic rickets
